# Multi-omics characterization and machine learning of lung adenocarcinoma molecular subtypes to guide precise chemotherapy and immunotherapy

**DOI:** 10.3389/fimmu.2024.1497300

**Published:** 2024-11-28

**Authors:** Yi Zhang, Yuzhi Wang, Haitao Qian

**Affiliations:** ^1^ Department of Laboratory Medicine, Guang’an People’s Hospital, Guang’an, Sichuan, China; ^2^ Key Laboratory of Clinical Laboratory Diagnostics (Ministry of Education), College of Laboratory Medicine, Chongqing Medical University, Chongqing, China; ^3^ Department of Laboratory Medicine, Deyang People’s Hospital, Deyang, Sichuan, China; ^4^ Pathogenic Microbiology and Clinical Immunology Key Laboratory of Deyang City, Deyang People’s Hospital, Deyang, Sichuan, China; ^5^ Department of Anesthesiology, The First People’s Hospital of Lianyungang, Lianyungang, Jiangsu, China

**Keywords:** lung adenocarcinoma, machine learning, multi-omics analysis, prognosis, treatment

## Abstract

**Background:**

Lung adenocarcinoma (LUAD) is a heterogeneous tumor characterized by diverse genetic and molecular alterations. Developing a multi-omics-based classification system for LUAD is urgently needed to advance biological understanding.

**Methods:**

Data on clinical and pathological characteristics, genetic alterations, DNA methylation patterns, and the expression of mRNA, lncRNA, and microRNA, along with somatic mutations in LUAD patients, were gathered from the TCGA and GEO datasets. A computational workflow was utilized to merge multi-omics data from LUAD patients through 10 clustering techniques, which were paired with 10 machine learning methods to pinpoint detailed molecular subgroups and refine a prognostic risk model. The disparities in somatic mutations, copy number alterations, and immune cell infiltration between high- and low-risk groups were assessed. The effectiveness of immunotherapy in patients was evaluated through the TIDE and SubMap algorithms, supplemented by data from various immunotherapy groups. Furthermore, the Cancer Therapeutics Response Portal (CTRP) and the PRISM Repurposing dataset (PRISM) were employed to investigate new drug treatment approaches for LUAD. In the end, the role of SLC2A1 in tumor dynamics was examined using RT-PCR, immunohistochemistry, CCK-8, wound healing, and transwell tests.

**Results:**

By employing multi-omics clustering, we discovered two unique cancer subtypes (CSs) linked to prognosis, with CS2 demonstrating a better outcome. A strong model made up of 17 genes was created using a random survival forest (RSF) method, which turned out to be an independent predictor of overall survival and showed reliable and impressive performance. The low-risk group not only had a better prognosis but also was more likely to display the “cold tumor” phenotype. On the other hand, individuals in the high-risk group showed a worse outlook and were more likely to respond positively to immunotherapy and six particular chemotherapy medications. Laboratory cell tests demonstrated that SLC2A1 is abundantly present in LUAD tissues and cells, greatly enhancing the proliferation and movement of LUAD cells.

**Conclusions:**

Thorough examination of multi-omics data offers vital understanding and improves the molecular categorization of LUAD. Utilizing a powerful machine learning system, we highlight the immense potential of the riskscore in providing individualized risk evaluations and customized treatment suggestions for LUAD patients.

## Introduction

In 2020, lung cancer (LC) was the second most commonly diagnosed cancer and the leading cause of cancer-related deaths, accounting for approximately 11.4% of all cancer cases and 18% of cancer-related fatalities (1). LC is primarily divided into two histological types: non-small cell lung cancer (NSCLC), representing about 85% of cases, and small cell lung cancer (SCLC), comprising roughly 15% of cases (2). NSCLC encompasses large-cell lung carcinoma (LCLC), lung adenocarcinoma (LUAD), and lung squamous cell carcinoma (LUSC), with LUAD being the most common form (3). Even with multiple treatment options like surgery, chemoradiotherapy, targeted therapy, and immunotherapy, the outlook for LUAD patients is still bleak, with just a 16% overall survival rate over five years (4). Although targeting EGFR, ALK, and TKI has shown potential in improving patient outcomes, drug resistance remains a significant challenge, leading to suboptimal therapeutic results (5, 6). Therefore, exploring novel biomarkers with high specificity and sensitivity is crucial for accurate diagnosis, personalized treatment, and precise prognosis prediction in LUAD.

Cancer is an extremely diverse and intricate illness, where individuals with identical histopathological categories may show different genetic mutations (7). Consequently, customized strategies for prevention, diagnosis, and treatment ought to be adapted to the clinical and omics characteristics of each patient (8). In the case of LUAD, molecular test results, including KRAS, EGFR, and TP53 mutations, as well as PDL1 expression, are utilized to assess prognosis (9–12). Integrating clinical and omics data for cancer prognosis can significantly improve predictive accuracy. Nevertheless, relying on individual omics datasets may result in the loss of critical genetic information, making it challenging to identify key pathogenic genes that reflect the diverse influencing factors present in the original sequencing data (13). Over the past few years, an increasing number of scientists have conducted comprehensive analyses of diverse omics datasets, achieving notable findings (14, 15). However, most prognostic studies on LUAD remain confined to a single omics dataset (16, 17). Moreover, the few studies that have attempted to combine multiple omics datasets with clinical data have struggled to do so effectively.

This research employed the MOVICS algorithm to merge DNA methylation patterns, genetic alterations, and data from mRNA, long non-coding RNA (lncRNA), and microRNA (miRNA), forming an extensive consensus subtype of LUAD. Subsequently, we pinpointed 32 genes linked to stable prognosis (SPRGs) through differential expression analysis among various subtypes and utilized 10 machine learning techniques, as well as 101 algorithmic combinations, to create a riskscore using four separate public datasets. This riskscore reliably predicts the prognosis of LUAD patients and assesses their responsiveness to chemotherapy and immunotherapy. To sum up, the riskscore obtained from various molecular subtypes offers new perspectives on prognosis, therapeutic targets, and the fundamental mechanisms for patients with LUAD.

## Materials and methods

### Data collection and preprocessing

**Figure 1:**
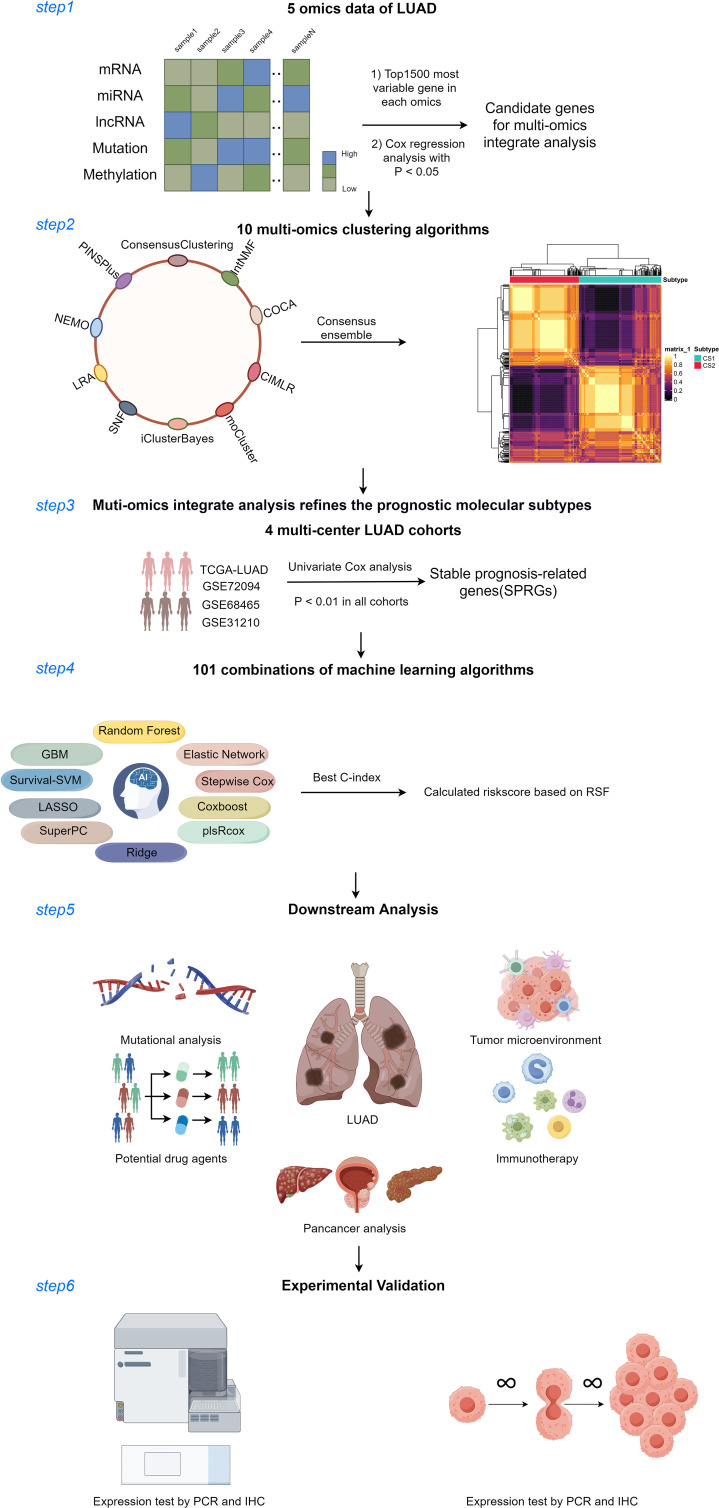
Diagram of analytic workflow in this study. The Diagram was drawn from the figdraw. (https://www.figdraw.com/static/index.html).

LUAD multi-omics data were downloaded from the TCGA database (https://portal.gdc.cancer.gov), encompassing clinical details (n=503), full transcriptome expression (FPKM format, n=576), DNA methylation (Methylation450k format, n=492), somatic mutations (mask format, n=526), and copy number variations (gistic2 format, n=516). Gene categories and names (lncRNA and mRNA) were annotated using official website files. The mature miRNA IDs from TCGA were identified using the “miRBaseVersions.db” package, while somatic mutations were analyzed with the “maftools” package. For DNA methylation data, β-values were logit-transformed, adjusted using ComBat, and then reverse logit-transformed (n=503). In addition, three other LUAD cohorts (GSE72094 (n=442), GSE68465 (n=462), and GSE31210 (n=246)) obtained from the Gene Expression Omnibus (http://www.ncbi.nlm.nih.gov/geo) were served as external validation cohorts. Gene symbols were assigned to the microarray data probe IDs based on the GPL15048, GPL96, and GPL570 platforms, and the expression profiles were deduplicated and normalized using the robust multichip average (RMA) algorithm (18). For genes associated with several probes, the average expression value was utilized. To eliminate potential batch effects across datasets, the ComBat function from the “sva” R package was applied (19). LUAD patients with OS information and shared gene expression data across all cohorts were included in this study. The number of patients included in each cohort is as follows: TCGA (n=503), GSE72094 (n=398), GSE68465 (n=442), and GSE31210 (n=226). **Supplementary Table 1** offers an overview of survival data and clinical characteristics for the patients. Notably, for multi-omics data analysis within the TCGA cohort, only patients with shared multi-omics profiles were selected as study subjects. This research flow chart is summarized in **Figure 1**.

### Integrative clustering based on multi-omics profiles

The “MOVICS” R package was created to enable thorough multi-omics clustering and visualization for cancer classification research (20). For integrative clustering analysis, we utilized TCGA multi-omics data to create two distinct data matrices, where columns correspond to shared samples (n=429) and rows denote omics characteristics. At first, a univariate Cox regression analysis was performed to pinpoint elements linked to overall survival (OS), using the pertinent data mentioned earlier. Genes with a mutation frequency exceeding 10% were classified as mutated. To precisely identify the subtypes, the cluster prediction index (CPI) and the gap statistic were utilized to assess the optimal number of clusters (21). The best cluster count for the given data was identified by choosing the number that maximized the gap statistic and CPI. Subsequently, ten different clustering techniques were utilized to categorize patients into unique subgroups. A consensus-based categorization was subsequently applied to ensure the robust identification of each subtype.

### Specific molecular characteristics and stability of consensus subtypes

To quantify pathway activity like the EGFR network, immune-suppressed oncogenic pathways, and radiotherapy-anticipated pathways, a sample-based gene set variation analysis (GSVA) was conducted on each enriched pathway to determine patient-specific GSVA scores (22). Using the “Reconstruction of Transcriptional Regulatory Networks and Analysis of Regulons (RTN)” R package, we built transcriptional regulatory networks (regulons) that included 23 transcription factors (TFs) linked to activated or suppressed targets, along with 71 potential regulators connected to chromatin remodeling in cancer (23). Following this, the expression levels of immune checkpoint genes were analyzed among various subtypes, and the ESTIMATE algorithm was utilized to calculate stromal and immune scores for each sample (24). The MeTIL score for tumor-infiltrating lymphocytes was determined using standard procedures for DNA methylation. To assess the relative presence of immune cells, we employed single-sample gene set enrichment analysis (ssGSEA) using the “GSVA” R package. To confirm the consistency of subtypes, we initially verified the clustering outcomes with subtype-specific biomarkers in the other group. Subsequently, we evaluated the reliability of consensus clustering by contrasting it with the Nearest Template Prediction (NTP) and Partition Around Medoids (PAM) methods, conducting these analyses on both the training and test groups (20).

### Integrative machine learning algorithms constructed an optimal signature

The “limma” R package was utilized to examine the genes with varying expression levels (DEGs) between the two MOVICS subtypes (25). Prognosis-related DEGs, identified as SPRGs, were determined in the training sets through univariate Cox regression (P<0.01). Following this, a comprehensive analysis was performed to develop a unified signature by employing 10 machine-learning techniques and 101 algorithm combinations for SPRGs. These included random survival forest (RSF), elastic network (Enet), Lasso, Ridge, stepwise Cox, CoxBoost, partial least squares regression for Cox (plsRcox), supervised principal components (SuperPC), generalized boosted regression modeling (GBM), and survival support vector machine (survival-SVM). The hyperparameters of all algorithms used the developer’s default settings. The procedure for generating the model included the following steps: (1) Prognostic biomarkers were pinpointed using Univariate Cox regression on the TCGA dataset; (2) Next, 101 different algorithm combinations were employed to create prediction models within a leave-one-out cross-validation (LOOCV) setup in the TCGA dataset; (3) These models were subsequently validated with the GEO cohorts (GSE72094, GSE68465, and GSE31210); (4) For each model, Harrell’s concordance index (C-index) was computed across all TCGA and GEO datasets, and the model with the highest average C-index was deemed the best. Comparable machine learning algorithms have been employed in prior studies (26). Parameter tuning details for the R scripts used in this study are available on GitHub (https://github.com/Zaoqu-Liu/IRLS). The detailed procedures for model selection and construction are described in the **Supplementary Methods**. Using the developed prognostic model, we computed a riskscore for each patient, categorizing them into high- and low-risk groups dependented on the median riskscore from whole datasets. Additionally, the performance of the riskscore was compared with that of 58 published signatures for predicting patient prognosis. To determine prognostic risk factors for LUAD, both univariate and multivariate Cox regression analyses were performed, and a predictive nomogram was created using the “rms” package in R, incorporating riskscore and clinical features.

### Somatic mutation and copy number variation analysis

The “maftools” R package was employed to assess the mutation status of individuals and to analyze patterns of mutually exclusive or coexisting mutations (27). GISTIC2.0 identified and pinpointed recurrent focal somatic copy number alters (CNAs) from genotype data, using a threshold of ±0.3 for amplifications and deletions (28). The TCGA-LUAD cohort’s scores for fractions of genome altered (FGA), genome gained (FGG), and genome lost (FGL) were derived from copy number fragment data. Data for tumor mutational burden (TMB) and tumor neoantigen burden (TNB) were retrieved from the UCSC database.

### RiskScore linked to immune features of TIME and molecular traits

Charoentong et al. identified 28 signatures associated with immune cells in their research (29). The “GSVA” R package was utilized to conduct single-sample gene set enrichment analysis (ssGSEA) in order to measure enrichment scores for each gene set and sample. Additionally, immune cell infiltration abundances were assessed using three other methodologies: TIMER (30), MCP-counter (31) and ESTIMAT (31). Data on the activation stages of the seven-phase Cancer Immunity Cycle were sourced from the tracking tumor immunophenotype (TIP) database (32). Additionally, the tumor immune microenvironment (TIME) was defined by the presence of 35 immune checkpoint inhibitor (ICI) genes, as outlined in our prior research (26). TIME and metabolic signatures were also gathered from earlier research and calculated using GSVA (33). To investigate variations in 50 hallmark pathways among different risk categories, we performed GSVA enrichment analysis using the “GSVA” R package, with pathways obtained from MSigDB database (34). To confirm the crucial outcomes from the GSVA analyses, gene set enrichment analysis (GSEA) was utilized.

### Evaluation of immunotherapy and chemotherapy response

The SubMap technique, which operates without supervision, was utilized to evaluate the expression similarities among patients with different responses to immunotherapy. Greater similarity in expression profiles indicated a higher likelihood of similar clinical outcome (35). The TIDE framework (http://tide.dfci.harvard.edu/) was employed to predict the likelihood of tumor immune evasion by analyzing gene expression data from cancer specimens (36, 37). The IMvigor210, GSE103668, and GSE79671 datasets were analyzed to predict immunotherapy response, with the riskscore calculated for each dataset. Drug sensitivity profiles were created using data from the Cancer Therapeutics Response Portal (CTRP) and Profiling Relative Inhibition Simultaneously in Mixture (PRISM) databases, which include sensitivity information for more than 1,000 compounds (38, 39). Both databases report AUC values as indicators of drug sensitivity, where higher AUC values correspond to reduced sensitivity to specific compounds. Substances with over 20% of data missing were omitted from the inferential study (40).

### Cell culture and transfections

Human LUAD cell lines A549, H838, and human bronchial epithelial cells BEAS-2B were sourced from Procell from Procell (Wuhan, China). H838 cells were cultured in RPMI-1640 medium supplemented with 10% fetal bovine serum (FBS), whereas A549 and BEAS-2B cells were cultured in Dulbecco’s Modified Eagle Medium (DMEM) with 10% FBS. Every cell culture was maintained in a moist environment containing 37°C and 5% CO_2_. Cells were transfected with SLC2A1 siRNAs (Hanheng, Shanghai, China) utilizing Lipofectamine 3000, adhering to the provided guidelines. **Supplementary Table 2** contains the sequences for SLC2A1 siRNAs.

### RNA extracting and real time polymerase chain reaction

Total RNA was isolated from LUAD cells with the AG RNAex Pro Reagent (AG, Changsha, China) and subsequently converted into cDNA using the Reverse Transcription Kit Mix (Promega, Madison, Wisconsin, USA). cDNA amplification followed, employing SYBR Premix Ex Taq II (Promega, Wisconsin, USA). mRNA levels were quantified via qRT-PCR on the Roche LightCycler 480II, using the 2^-ΔΔCt^ method, with Beta-actin as the endogenous control. Primer sequences are provided in **Supplementary Table 3**.

### Immunohistochemical staining

Outdo Biotech (Shanghai, China) provided fifteen sets of LUAD tumor and nearby normal tissue microarrays (HLugA030PG04). Immunohistochemical staining was conducted on theses microarrays. The microarrays were incubated overnight at 4°C with rabbit anti-SLC2A1 antibodies (ABclonal, Wuhan, China, Cat. A6982, 1:100). The evaluation of SLC2A1 expression utilized a scoring method that considered staining intensity (0 for no staining, 1 for weak, 2 for moderate, and 3 for strong) and the proportion of cells showing positive staining (<5% = 0, 5% to <25% = 1, 25% to 50% = 2, >50% to <75% = 3, >75% = 4). The ultimate score was calculated by multiplying the extent rating with the intensity rating.

### Cell Counting Kit-8 (CCK-8) and flat plate clone formation assays

For the CCK-8 test, 10,000 cells per well were seeded in triplicate into 96-well plates and incubated at 37°C with 5% CO_2_ in a humidified atmosphere. The assay was conducted at 24, 48, and 72 hours post-seeding, following the manufacturer’s instructions. During the plate clone formation test, cells in the exponential growth phase were plated at a concentration of 500 cells per milliliter in 6-well dishes. The cells were cultured at 37°C in a humid atmosphere containing 95% air and 5% carbon dioxide for approximately two weeks. Colonies were then imaged and quantified using ImageJ software.

### Transwell invasion and migration assays

Transwell experiments were conducted to assess both cell migration and invasion. The transwell inserts were placed into a 24-well culture plate, with the insert referred to as the upper chamber and the plate as the lower compartment. Cells were broken down in a medium without serum, set to a concentration of 10^7^ cells per milliliter, and subsequently placed in the upper chamber. The bottom compartment was occupied by a base medium with 10% FBS. For invasion assays, the upper membrane was precoated with 40 µl of 8% matrigel matrix. Following a 24-hour period, the cells were treated with 4% paraformaldehyde, rinsed with PBS, stained using 0.1% crystal violet, and observed under an optical microscope at suitable magnification.

### Wound-healing assays

A 6-well plate was inoculated with a cell mixture at a concentration of 2 × 10^5^ cells per well. Once the cells grew more than 80% fusion, a wound was created using a 200 µl pipette tip. The cells were then washed twice with PBS and incubated in a serum-free culture medium for 24 hours. Wound closure was photographed at 24 hours using a light microscope, and cell migration ability was assessed by calculating the wound-healing rate.

### Statistical analysis

R version 4.3.0 was utilized for data handling, statistical computations, and visualization. To assess relationships between continuous variables, Pearson’s correlation coefficients were used, and the Wilcoxon test was applied for differential analysis. A p-value of less than 0.05 was considered indicative of statistical significance.

## Results

### Multi-omics consensus prognosis-related molecular subtypes of LUAD

**Figure 2:**
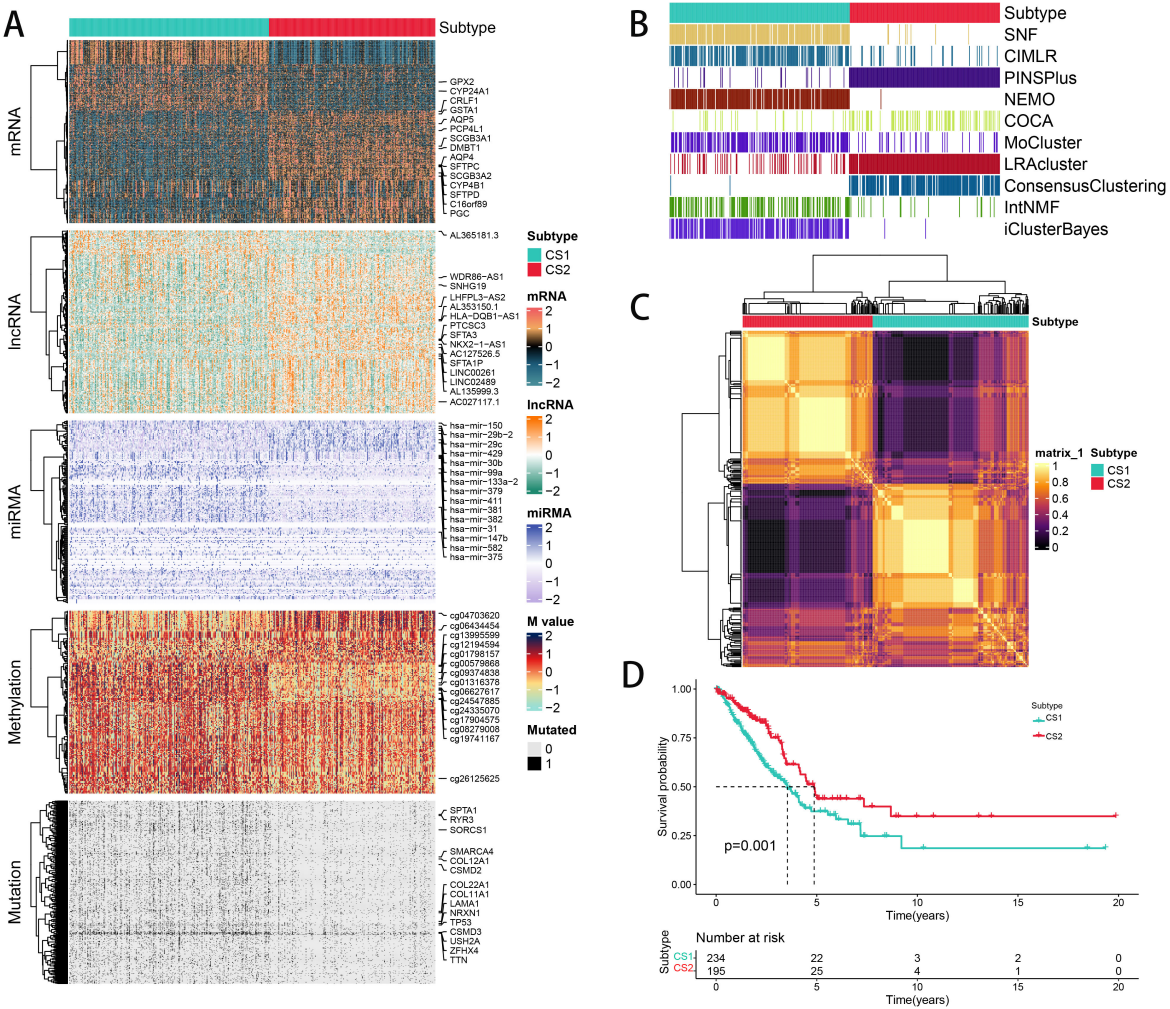
The multiomics integrative consensus subtypes of LUAD. **(A)** Comprehensive heatmap depicting consensus ensemble subtypes, featuring data on mRNA, lncRNA, miRNA, DNA CpG methylation sites, and mutant genes. **(B)** Clustering of LUAD patients using 10 advanced multiomics clustering methods. **(C)** Consensus matrix for three clusters derived from the 10 algorithms. **(D)** Survival outcomes comparison between the two subtypes.

We independently identified two cancer subtypes (CSs) using 10 different multi-omics ensemble clustering algorithms. The quantity of subtypes was established by combining multiple algorithms and findings from prior studies (**Supplementary Figures 1, 2**). The clustering results were then consolidated by a consensus ensemble approach using multi-omics data, with the top 15 items for each omic presented in **Figures 2A-C**. Notably, CS2 exhibited a significantly higher survival probability compared to CS1 (**Figure 2D**).

### Biological characteristics for CSs

**Figure 3:**
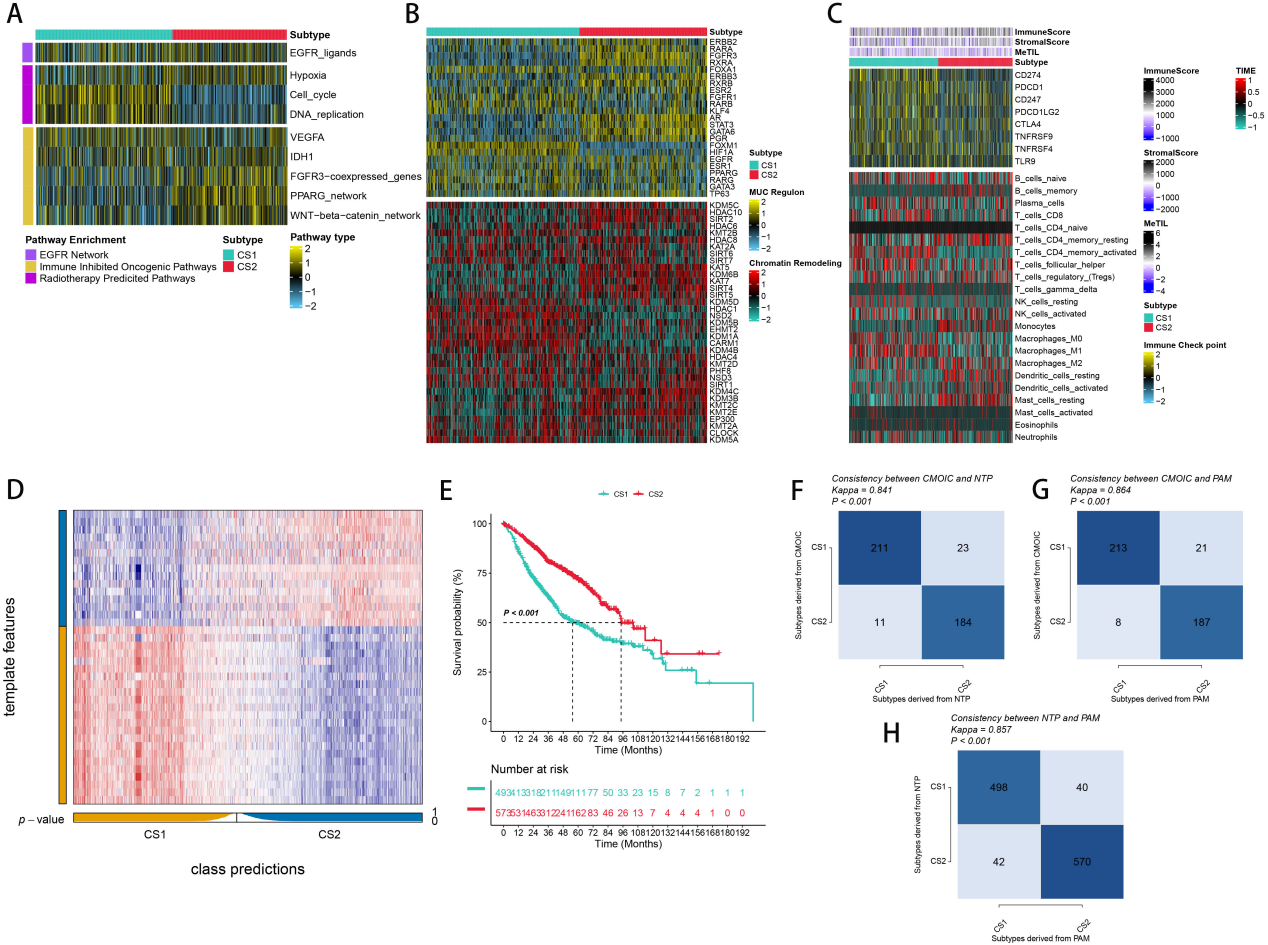
Molecular Landscape and Validation of LUAD Consensus Subtypes (CSs). **(A)** Enrichment of three subtypes with various treatment-related and bladder cancer-related signatures. **(B)** Activity profiles of 23 transcription factors (TFs) and potential regulators of chromatin remodeling across the three subtypes. **(C)** Immune profiles within the TCGA cohort, featuring a heatmap with annotations for immune and stromal enrichment scores, and DNA methylation of tumor-infiltrating lymphocytes. The heatmap includes expression levels of canonical immune checkpoint genes and enrichment levels of 24 tumor immune microenvironment-related immune cells. **(D)** Validation of LUAD CSs using the nearest template prediction in the META-LUAD cohort. **(E)** Survival analysis of LUAD CSs within the meta cohort. **(F, G)** Consistency of CSs with Nearest Template Prediction (NTP) and Prediction Analysis for Microarrays (PAM) in the TCGA cohort. **(H)** Consistency of NTP with PAM in the meta-cohort.

To explore the potential biological functions of the different subtypes, we characterized their molecular features. Using the ssGSEA algorithm, we assessed the enrichment of various molecular signatures within the samples. Notably, CS2 had a significant enrichment of immune-suppressive cancer pathways, while CS1 showed a higher presence of pathways indicative of radiotherapy effectiveness. This suggests that CS1 may be more responsive to radiotherapy, while CS2 shows a greater sensitivity to immunosuppressive therapy (**Figure 3A**). In order to delve deeper into transcriptomic variations, we examined possible regulators linked to cancer chromatin modification and 23 LUAD-specific transcription factors (TFs) (**Figure 3B**). The strong correlation between regulator activity and CSs underscores the biological significance of these subtypes. Specifically, ERBB2, RARA, FGFR, RXRA, ERBB3, RXRB, ARM, STAT3, GATA6, and PGR were markedly activated in CS2, while FOXA1, RARB, FOXM1, and HIF1A were activated in CS1. The activity profiles of regulons associated with cancer-related chromatin changes highlight possible patterns of varied regulation among subtypes, suggesting that transcriptional networks influenced by epigenetics could be crucial distinguishing elements between these molecular subtypes. Additionally, we quantified microenvironmental cell infiltration levels and observed a significant increase in immune cell infiltration in CS1 (**Figure 3C**). Through differential expression analysis among subtypes, we choosed 50 genes that are distinctly upregulated in each subtyp act as classifiers and confirmed their consistency across several external cohorts. Using the NTP technique, each sample from the external groups was categorized into one of the determined subtypes. In line with these results, CS2 in the meta cohorts showed improved outcomes, a pattern also seen in additional external cohorts (**Figures 3D, E**). We also assessed the concordance of subtype classifications using the NTP and PAM algorithms (**Figures 3F-H**).

### Integrative machine learning algorithms developed an optimal prognostic signature

**Figure 4:**
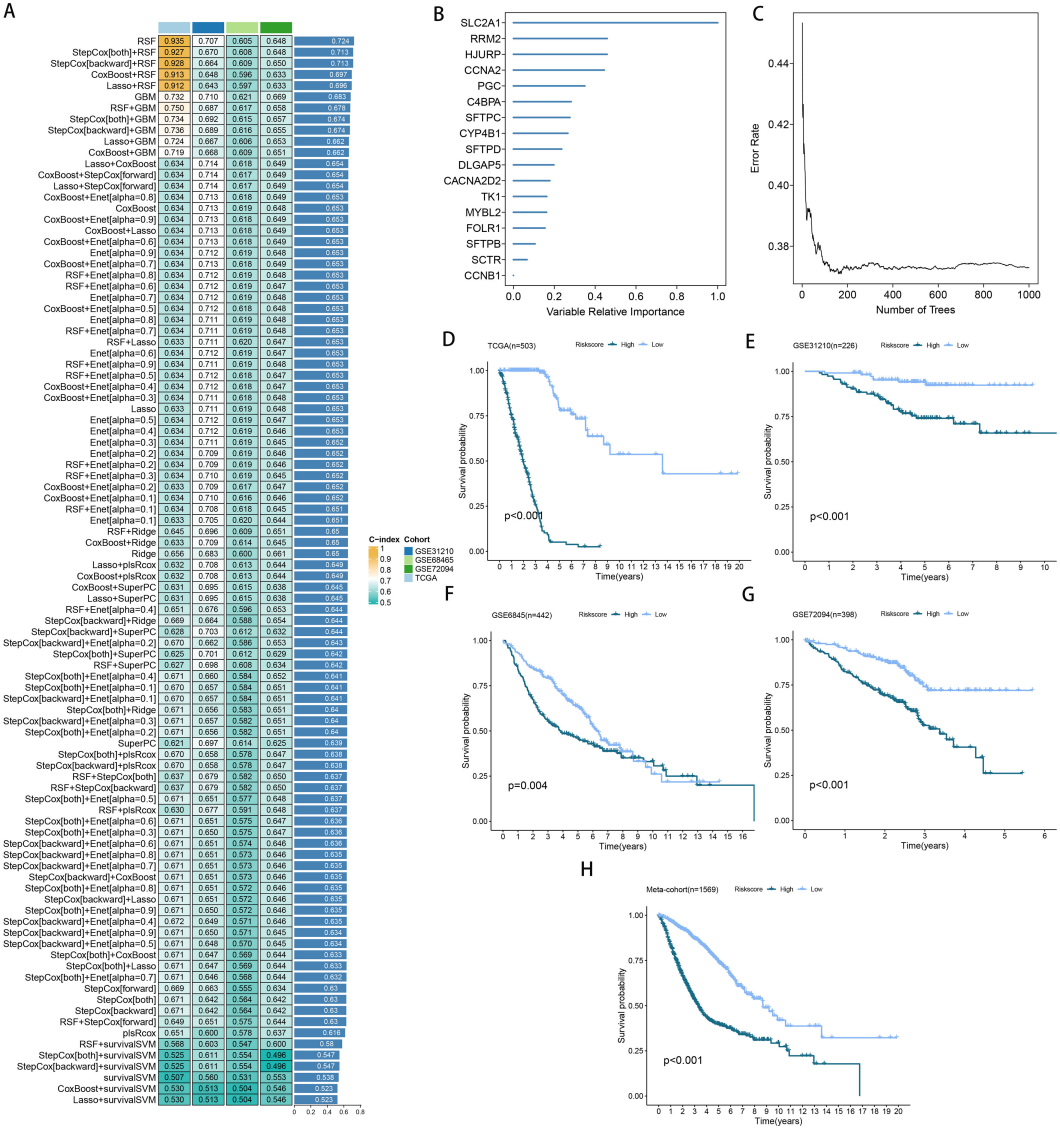
A model was established and validated through a machine learning-based integrative approach. **(A)** Utilizing 101 different machine learning algorithms, the optimal model was identified, and the concordance index (C-index) for each model was calculated across all cohorts. **(B, C)** Analysis of the number of trees required to achieve minimal error in the model and the significance of the 16 SPRGs using the Random Survival Forest (RSF) algorithm. **(D-H)** Kaplan-Meier survival curves depicting overall survival (OS) based on the risk score in TCGA, GSE31210, GSE68465, GSE72094, and a meta-cohort.

**Figure 5:**
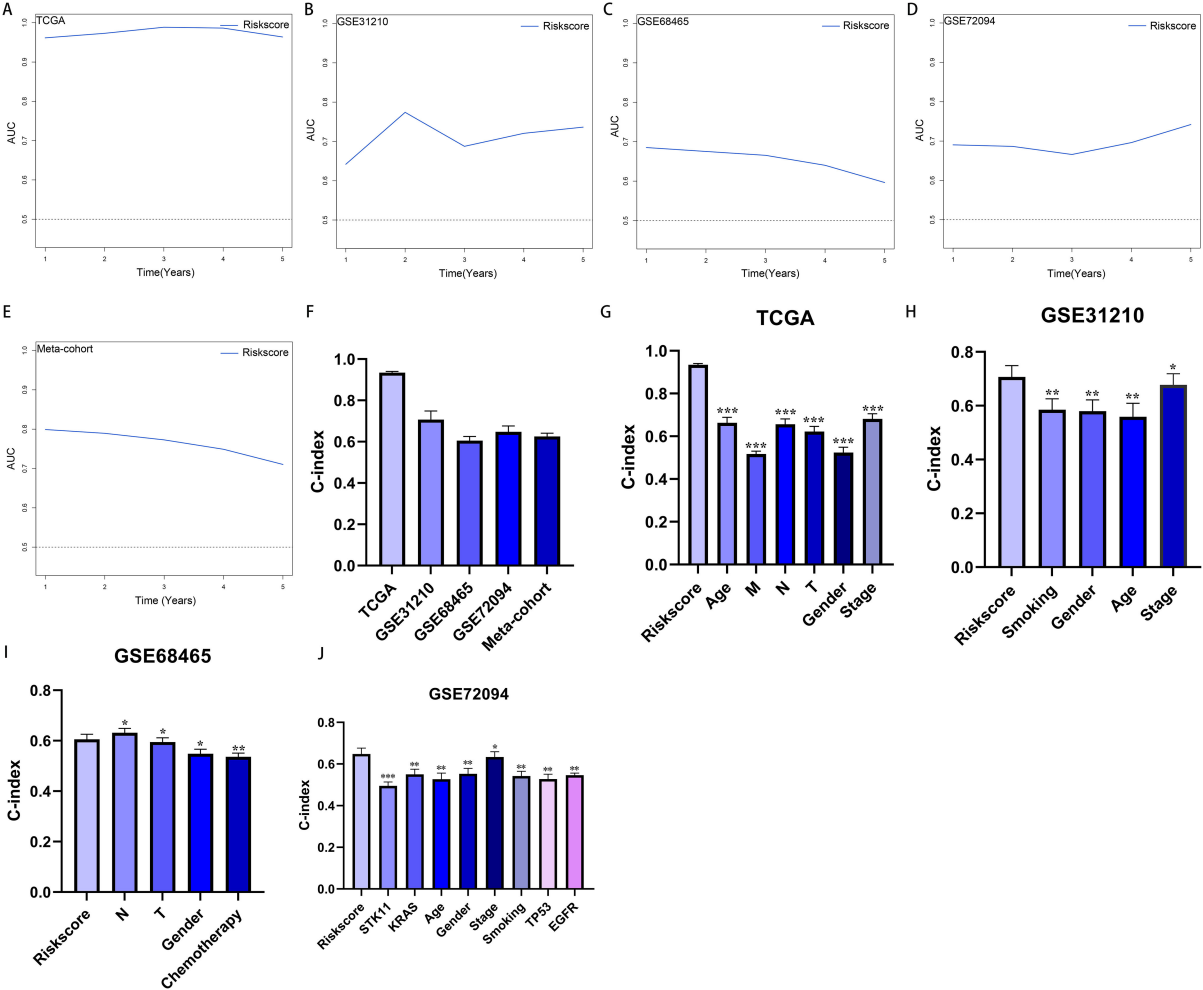
Evaluation of the riskscore. **(A–E)** Time-dependent receiver operating characteristic curve of riskscore for predicting the prognosis of LUAD patients from TCGA, GSE31210, GSE68465, GSE72094 and meta-cohort. **(F)** The C-index of the riskscore for the TCGA, GSE31210, GSE68465, GSE72094 cohorts. **(G–J)** The C-index of the riskscore and other clinical factors in the TCGA, GSE31210, GSE68465, GSE72094 cohorts. *P < 0.05, **P < 0.01, ***P < 0.001.

**Figure 6:**
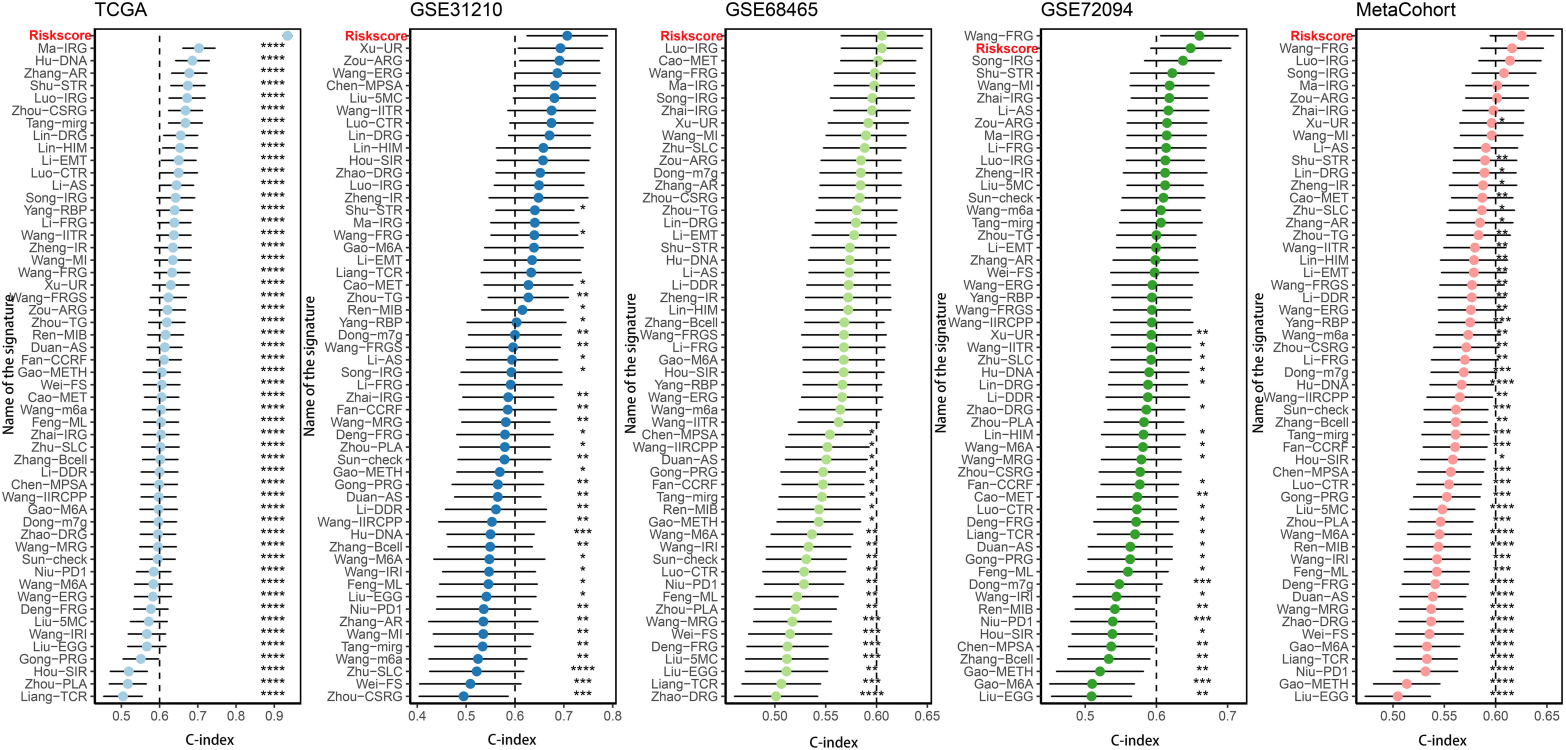
Comparison of riskscore and other gene expression-based prognostic signatures in LUAD based on the TCGA, GSE31210, GSE68465, GSE72094 and meta-cohort. *P < 0.05, **P < 0.01, ***P < 0.001, ****P < 0.0001.

More than 35 possible prognostic biomarkers underwent a comprehensive analysis employing ten machine learning techniques, enabling the creation of a precise and reliable prognostic model. A total of 101 predictive models were generated, and their C-index values for the training and testing groups are shown in **Figure 4A**. Out of all the models, the one built with the RSF technique was deemed the best, attaining the top average C-index of 0. 724 (see **Figure 4A**). Within the RSF framework, the best trees were achieved when the partial likelihood deviance hit its lowest point (**Figures 4B, C**). Seventeen genes were ultimately selected and used to construct the model. Kaplan-Meier analysis of the training group revealed that individuals identified as low-risk experienced notably improved outcomes compared to those labeled as high-risk (**Figure 4D**). This trend was also consistently seen in the testing and meta groups (**Figures 4E-H**). **Supplementary Figure 3** demonstrates significant survival disparities between high- and low-risk groups across different subcategories such as age, gender, T, N, M, and stage. The outcomes of the chi-squared analysis reveal a notable link between the riskscore and clinical features like status, stage, N, and T (**Supplementary Figure 4**). To further validate the prognostic significance of the genes included in the riskscore, we conducted a Kaplan-Meier analysis across pan-cancer datasets, which produced results largely consistent with those derived from the Cox algorithm. Additionally, These genes exhibited significant associations with the majority of tumors, highlighting their strong prognostic relevance for cancer patients (**Supplementary Figure 5**). In the TCGA cohort, the AUC for predictions spanning 1 to 5 years varied between 0.94 and 0.99 (**Figure 5A**), indicating that the prognostic model successfully distinguished between positive and negative outcomes for LUAD patients. The signature’s predictive accuracy stayed consistent and similar across both the testing and meta cohorts (**Figures 5B-E**). Notably, the C-index of the riskscore surpassed that of nearly all clinical characteristics in both the training and testing cohorts (**Figures 5F-J**). To evaluate the predictive power of the riskscore relative to other prognostic signatures, we randomly selected 58 previously established LUAD prognostic signatures (**Supplementary Table 2**) and calculated their C-index values. As illustrated in **Figure 6**, our riskscore’s C-index surpassed that of the majority of other signatures in the training, testing, and meta cohorts. Both univariate and multivariate Cox regression analyses determined that the riskscore independently influences LUAD patient outcomes in the training and testing groups (refer to **Tables 1**–**4**).

**Table 1:** Univariate and multivariate Cox analysis of the clinicopathological features and riskscore with OS for TCGA cohort.

Characteristics	Univariate Cox		Multivariate Cox	
	HR (95%CI)	*P* value	HR (95%CI)	*P* value
Stage	1.977 (1.586-2.463)	**< 0.001**	1.374 (0.956-1.974)	0.086
N	1.942 (1.575-2.394)	**< 0.001**	1.262 (0.947-1.681)	0.113
T	1.816 (1.386-2.38)	**< 0.001**	1.131 (0.801-1.598)	0.484
Age	1.038 (0.822-1.31)	0.754	NA	NA
Sex	1.041 (0.847-1.28)	0.7	NA	NA
M	1.727 (1.18-2.527)	**0.005**	0.906 (0.57-1.44)	0.676
Riskscore	0.106 (0.075-0.149)	**< 0.001**	0.133 (0.09-0.197)	**< 0.001**

Significant value is given in bold.

**Table 2:** Univariate and multivariate Cox analysis of the clinicopathological features and riskscore with OS for GSE68465 cohort.

Characteristics	Univariate Cox		Multivariate Cox	
	HR (95%CI)	*P* value	HR (95%CI)	*P* value
N	2.029 (1.689-2.438)	**< 0.001**	1.906 (1.568-2.318)	**< 0.001**
T	2.062 (1.587-2.68)	**< 0.001**	1.815 (1.373-2.4)	**< 0.001**
Gender	1.262 (1.051-1.516)	**0.013**	1.257 (1.035-1.527)	**0.021**
chemotherapy	1.412 (1.15-1.734)	**< 0.001**	1.319 (1.062-1.639)	**0.012**
Riskscore	0.728 (0.607-0.875)	**< 0.001**	0.824 (0.679-0.999)	**0.039**

Significant value is given in bold.

**Table 3:** Univariate and multivariate Cox analysis of the clinicopathological features and riskscore with OS for GSE31210 cohort.

Characteristics	Univariate Cox		Multivariate Cox	
	HR (95%CI)	*P* value	HR (95%CI)	*P* value
smoking	1.417 (0.882-2.277)	0.15	NA	NA
gender	1.344 (0.839-2.152)	0.219	NA	NA
age	1.263 (0.777-2.052)	0.346	NA	NA
stage	2.774 (1.732-4.441)	**< 0.001**	2.132 (1.298-3.502)	**0.003**
Riskscore	0.286 (0.153-0.532)	**< 0.001**	0.456 (0.245-0.846)	**0.013**

Significant value is given in bold.

**Table 4:** Univariate and multivariate Cox analysis of the clinicopathological features and riskscore with OS for GSE72094 cohort.

Characteristics	Univariate Cox		Multivariate Cox	
	HR (95%CI)	*P* value	HR (95%CI)	*P* value
STK11	1.028 (0.72-1.469)	0.879	NA	NA
KRAS	0.767 (0.588-0.999)	**0.049**	0.909 (0.692-1.194)	0.494
Age	1.258 (0.836-1.894)	0.27	NA	NA
Gender	0.733 (0.564-0.952)	**0.02**	0.689 (0.526-0.901)	**0.007**
Stage	1.969 (1.477-2.625)	**< 0.001**	2.006 (1.497-2.687)	**< 0.001**
Smoking	1.248 (0.694-2.245)	0.459	NA	NA
TP53	0.861 (0.645-1.151)	0.313	NA	NA
EGFR	2.58 (1.274-5.226)	**0.008**	1.989 (0.969-4.079)	0.061
Riskscore	0.575 (0.438-0.755)	**< 0.001**	0.531 (0.398-0.708)	**< 0.001**

Significant value is given in bold.

Afterward, we utilized the GSCALite public platform (http://bioinfo.life.hust.edu.cn/web/GSCALite/) to comprehensively examine the multi-omics characteristics of the riskscore across 31 different cancer types in the TCGA dataset. This study found that in cancer types with over 12 tumor and para-cancer tissues, the genes RRM2, TK1, CCNB1, DLGAP5, CCNA2, MYBL2, and HJURP were repeatedly overexpressed across various cancer tissues (**Supplementary Figure 6A**). Furthermore, we noticed a positive relationship between mRNA expression levels and CNVs of riskscore genes in the majority of cancer types (**Supplementary Figure 6B**). Further examination of CNV frequency variations revealed notable disparities in the CNVs of riskscore genes across different cancer types (**Supplementary Figures 6C, D**). Additionally, we noticed that the methylation levels of riskscore genes varied considerably between cancerous and normal tissues in the majority of cancer samples (**Supplementary Figure 7A**). Additionally, the methylation levels of these genes were inversely correlated with their mRNA expression levels across most cancers (**Supplementary Figure 7B**). Furthermore, riskscore genes were found to activate the pan-cancer cell cycle while significantly inhibiting the hormone ER pathway (**Supplementary Figures 7C, D**).

### Correlation between genomic alterations and the riskscore

**Figure 7:**
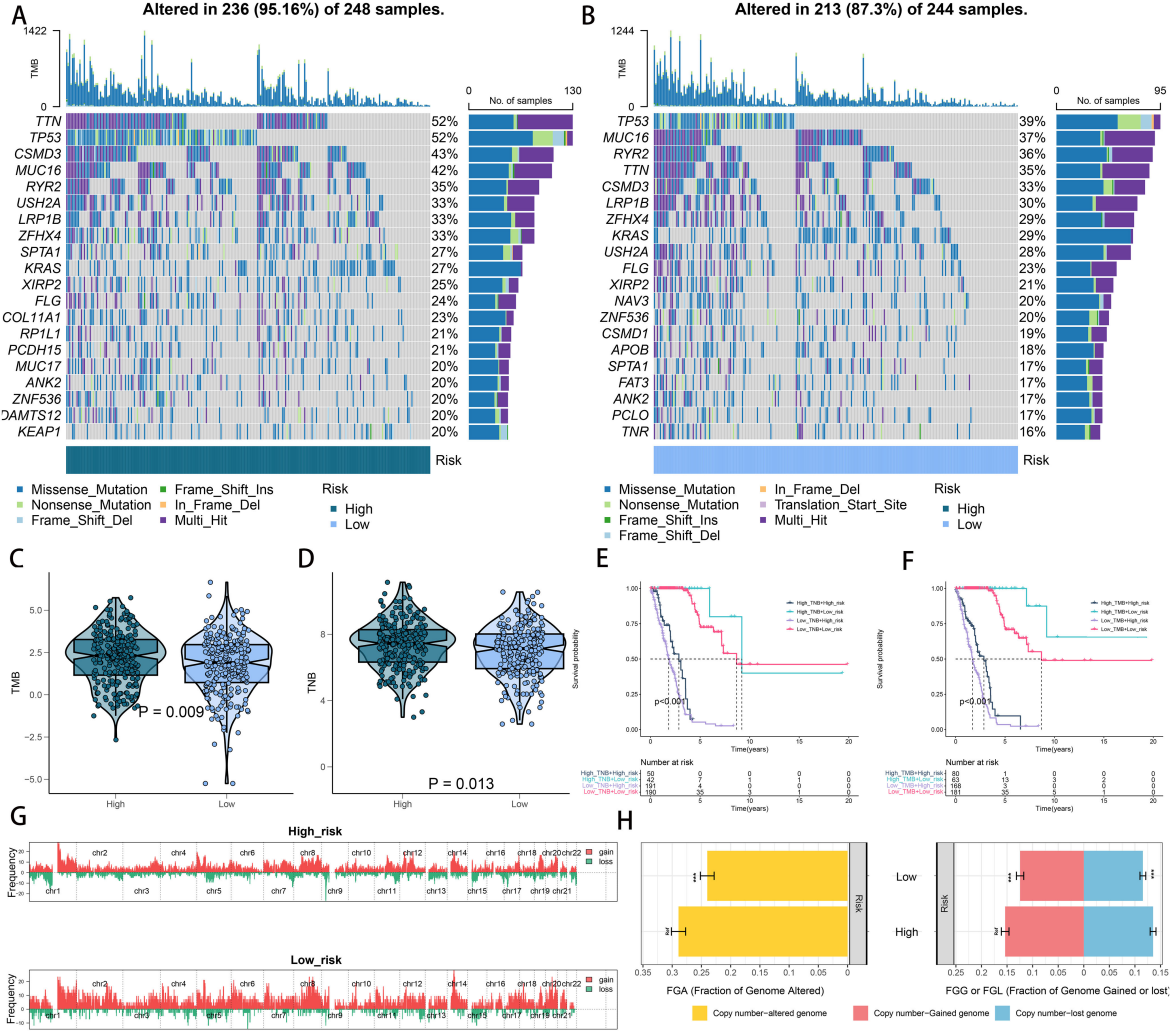
Integrated comparisons of somatic mutation and CNVs between high- and low-risk groups in the TCGA cohort. **(A, B)** Waterfall plots showing the mutation information of the top 20 genes with the highest mutation frequency in the risk groups. **(C, D)** Distribution of TMB and TNB in the high- and low-risk groups. **(E)** Kaplan–Meier curves for patients stratified by both TMB and riskscore. **(F)** Kaplan–Meier curves for patients stratified by both TMB and riskscore. **(G)** Gene fragments profiles with amplification red and deletion green among the high- and low-risk groups. **(H)** Comparison of the fraction of the genome altered, lost, and gained between the different risk groups. ***P < 0.001.

Examining somatic mutations and CNVs uncovered notable distinctions between the low- and high-risk categories. We charted the 20 genes with the highest mutation frequencies across both risk categories, pinpointing TP53, TTN, MUC16, CSMD3, and RYR2 as the leading five based on mutation rates (**Figures 7A, B**). **Figures 7C, D** illustrate that the TNB and TMB levels were markedly elevated in the high-risk group relative to the low-risk group. According to Kaplan-Meier analysis, patients grouped by TNB and riskscore showed that individuals with elevated TNB and low risk had the most favorable prognosis, while those with reduced TNB and high risk had the poorest outcome (**Figure 7E**). A comparable trend was observed when integrating TMB with riskscore; patients with elevated TMB and minimal risk had the most favorable prognosis, whereas those with reduced TMB and high risk experienced the worst outcomes (**Figure 7F**). Analysis of the somatic mutation spectrum showed that the high-risk group had increased numbers of synonymous and non-synonymous mutations, along with a higher overall mutation count, in comparison to the low-risk group (**Supplementary Figures 8A–C**). Additionally, an increase in mutations was positively linked to the riskscore (**Supplementary Figures 8A–C**). Importantly, 27 genes exhibited markedly different mutation rates between the two cohorts, with a notable presence of co-mutations (**Supplementary Figures 8D, E**). Analyzing CNV between the two risk groups revealed a greater occurrence of CNV events in the high-risk group (**Figure 7G**). These findings were further corroborated by the FGA, FGG, and FGL rates (**Figure 7H**).

### RiskScore correlated with immune characters of TIME and molecular characteristics

**Figure 8:**
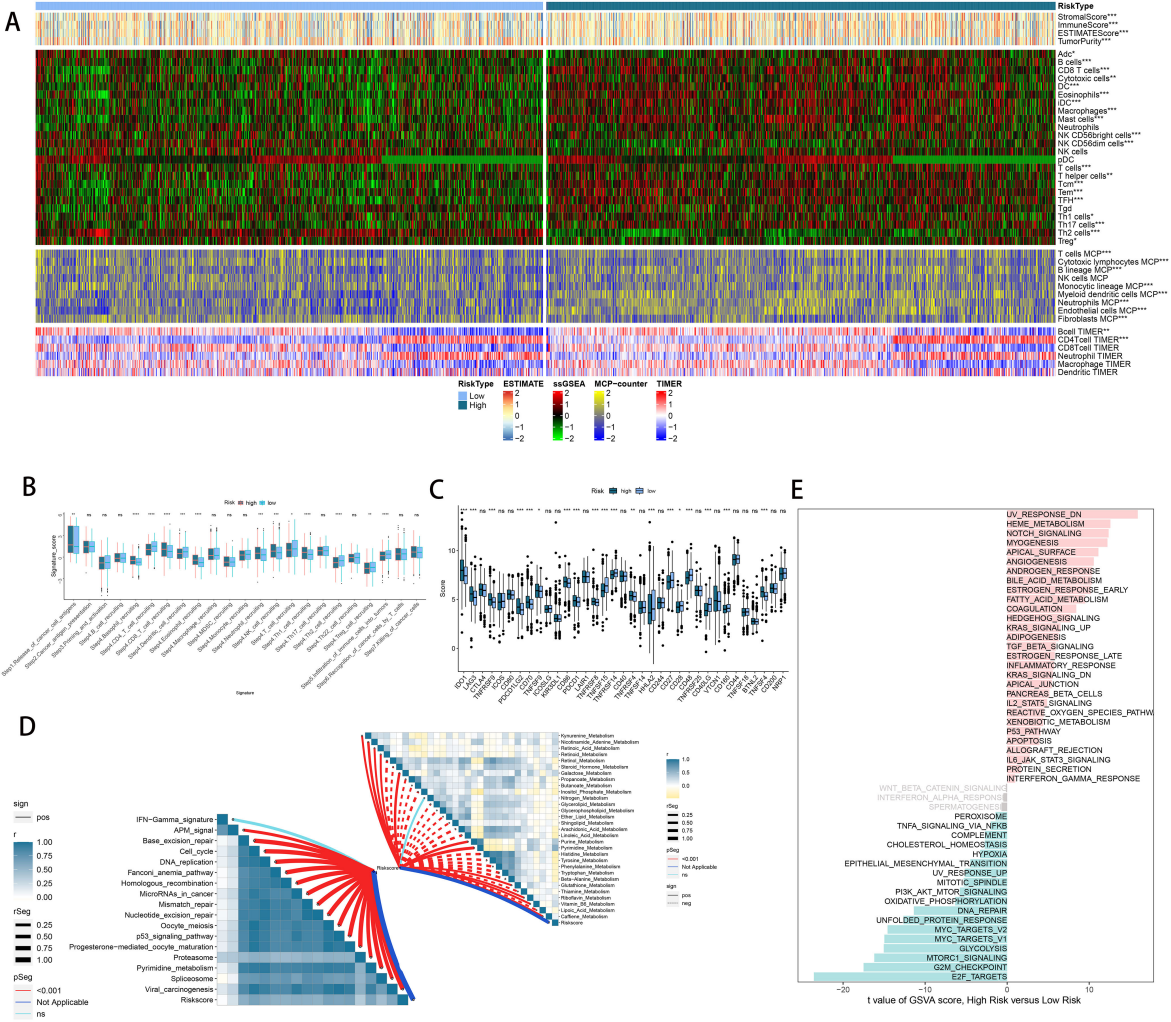
Immune-related characteristics of the riskscore. **(A)** Heatmap displaying the correlation between the riskscore and immune infiltrating cells in the meta cohort. **(B)** Boxplot showing the differences of anti-cancer immunity score between different risk groups. **(C)** Comparison of immune checkpoint-related genes levels between different risk groups in the meta-cohort. **(D)** The correlations between the riskscore and immune-related pathways, metabolic pathways based on GSVA of GO and KEGG terms were displayed in butterfly plot. **(E)** The difference in the hallmark gene sets between different risk groups. ns, not significant, *P < 0.05, **P < 0.01, ***P < 0.001, ****P < 0.0001.

As previously noted, we assessed the infiltration levels of diverse immune cells in LUAD using various TIME contexture decoding algorithms. Among the high-risk cohort, TIME exhibited notably increased immune cell infiltration relative to the low-risk cohort (**Figure 8A**). In the high-risk group, the cancer immune cycle exhibited greater dynamism, highlighted by elevated activities like the release of antigens from tumor cells and the augmented recruitment of basophils, CD8 T cells, neutrophils, and natural killer (NK) cells (**Figure 8B**). Additionally, the riskscore was positively correlated with the levels of various immune checkpoints like CD274, CTLA4, and PDCD1, along with the enrichment scores of gene signatures linked to immunotherapy effectiveness (**Figure 8C**). Conversely, the riskscore showed a negative correlation with many metabolic pathways (**Figure 8D**). **Figure 8E** illustrates that the high-risk group showed notable enrichment in pathways associated with tumor progression, including PI3K-AKT-MTOR signaling, MYC targets, MTORC1 signaling, and the G2M checkpoint. These findings were further confirmed by GSEA analysis (**Supplementary Figure 9**).

### Riskscore-based treatment strategy for LUAD

**Figure 9:**
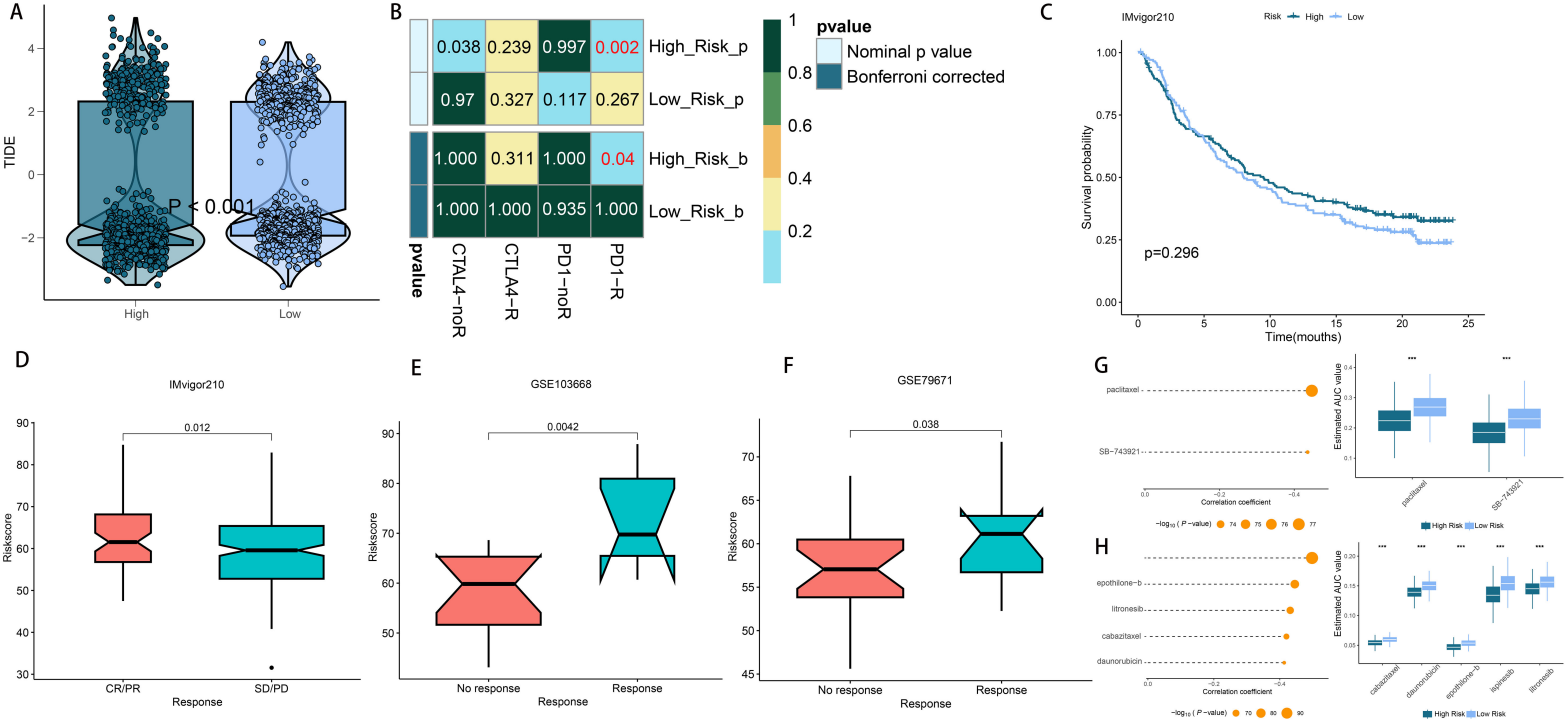
Differential putative immunotherapy and chemotherapy response for patients from high- and low-risk groups. **(A)** Violin plot showing different TIDE scores from patients in different risk group. **(B)** Submap analysis of the meta-cohort and melanoma patients with detailed immunotherapeutic information. **(C)** Kaplan-Meier curve for patients in high- and low-risk groups in the IMvigor210 cohort **(D–F)** Box plot showing different riskscore from patients with immunotherapy responses in the IMvigor210, GSE103668 and GSE79671 cohorts. **(G)** The results of correlation analysis and differential drug response analysis of CTRP-derived drugs. **(H)** The results of correlation analysis and differential drug response analysis of PRISM-derived drugs. ***P < 0.001.

Considering the high rate of genomic changes and tumor mutational burden in high-risk LUAD patients, along with their active TIME and elevated levels of immune checkpoint molecules, we proposed that these individuals could show greater responsiveness to immunotherapy. In support of this, the TIDE online tool showed that the low-risk group had notably reduced TIDE scores (**Figure 9A**), and Submap analysis demonstrated that the gene expression patterns of low-risk individuals were more similar to those of melanoma patients who responded positively to immune checkpoint inhibitors (ICIs) (**Figure 9B**). These findings suggest that patients with a low riskscore may derive greater benefit from immunotherapy. Furthermore, we assessed the prognostic significance of the riskscore across three immunotherapy datasets. Importantly, there were no notable differences in survival rates between the high- and low-risk groups in the IMvigor210 study (**Figure 9C**). However, an analysis across the IMvigor210, GSE103668, and GSE79671 cohorts demonstrated that a higher riskscore was associated with increased immunotherapy response rates (**Figures 9D-F**). Additionally, we utilized a formula to pinpoint agents that might be effective for the high-risk group, leading to the identification of two agents from CTRP (paclitaxel and SB-743921) and four from PRISM (epothilone-b, litronesib, cabazitaxel, and daunorubicin). **Figures 9G, H** illustrate that the predicted AUC values for these agents have a statistically significant inverse relationship with the riskscore.

### Validation of riskscore in human tissues and pan-cancer

**Figure 10:**
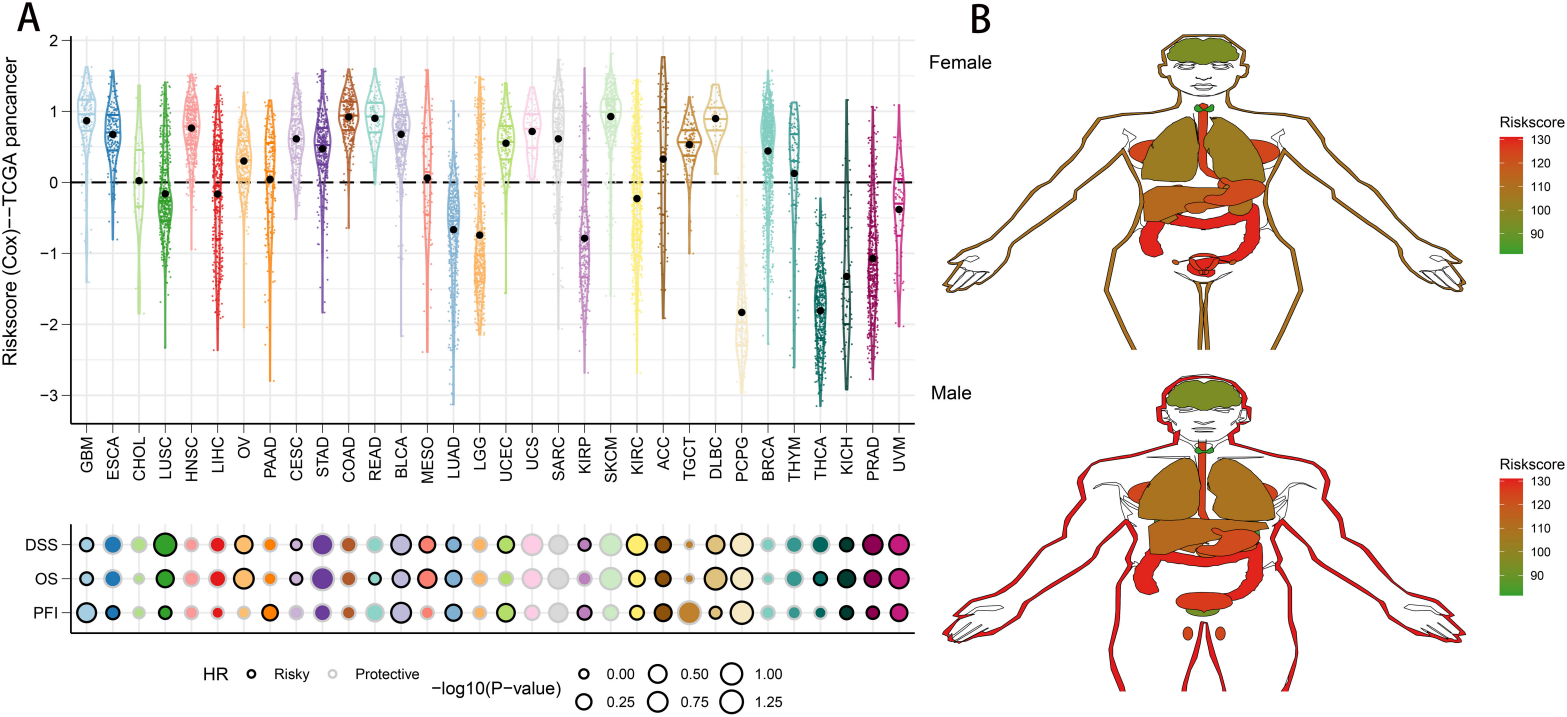
Predictive accuracy of the riskscore in the TCGA-pancancer set. **(A)** Distribution and predictive value of riskscore in solid tumors in the TCGA-pancancer set. **(B)** Differences in the distribution of riskscore in tumor tissues in different organs.

To evaluate the generalizability of the riskscore across various tumor types, we applied the same model to pan-cancer data. Using the defined formula, we derived the distribution of the riskscore across multiple cancers, with skin cutaneous melanoma displaying the highest riskscore (**Figure 10A**). The riskscore was also identified as a major risk factor in various cancers, such as glioma, lung squamous cell carcinoma, kidney renal clear cell carcinoma, bladder cancer, adrenocortical carcinoma, diffuse large B-cell lymphoma, pheochromocytoma and paraganglioma, kidney chromophobe, prostate adenocarcinoma, and uveal melanoma (**Figure 10A**). Furthermore, we assessed the riskscore in 31 different tumor tissues. Analysis revealed that males had significantly higher riskscores in esophageal, stomach, colon, gallbladder, ovarian, and uterine cancers. Conversely, in females, elevated riskscore were observed in skin, esophagus, stomach, colon, gallbladder, prostate, and testicular cancers (**Figure 10B**). This gender-based variation in riskscore profiles underscores the need for tailored approaches in cancer risk assessment and management.

### Inhibition of cell proliferation and migration by SLC2A1 knockdown

**Figure 11:**
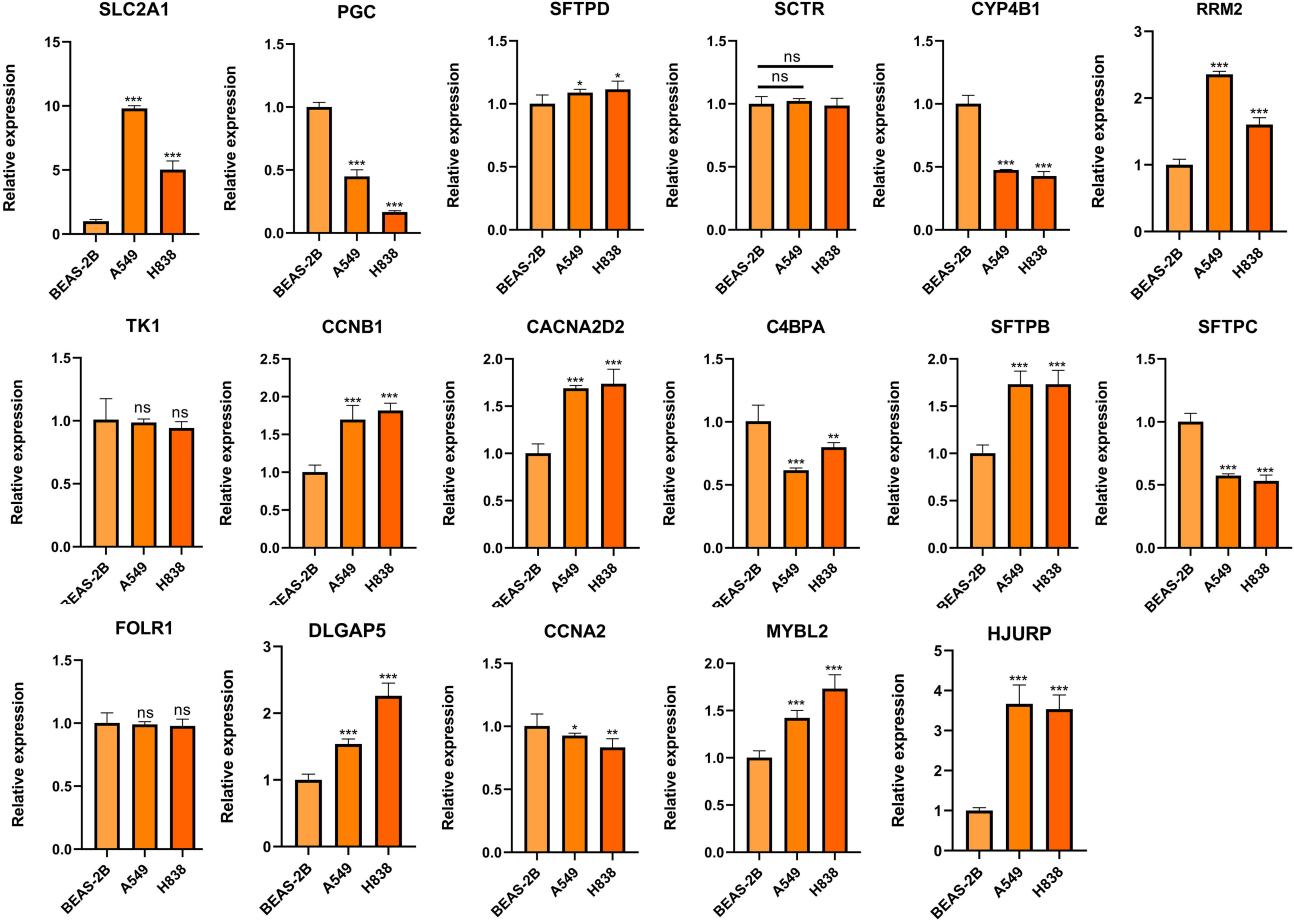
Validation of the expression of genes comprising the riskscore in LUAD and lung epithelial cells. ns, not significant. *P < 0.05, **P < 0.01, ***P < 0.001.

**Figure 12:**
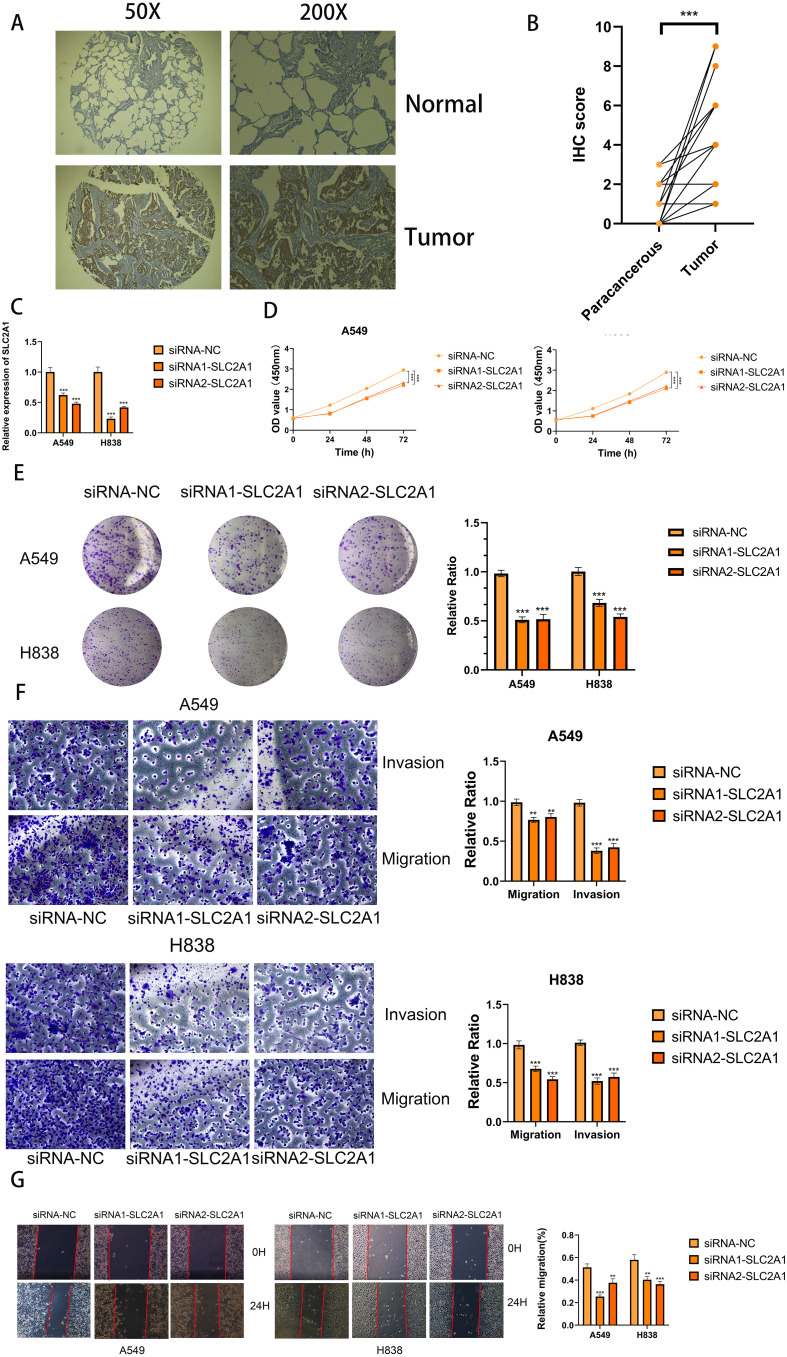
SLC2A1 promoted proliferation, migration, and invasion of LUAD cell lines. **(A, B)** Immunohistochemistry (IHC) analysis of SLC2A1 in 15 LUAD and 15 adjacent tissues. **(C)** Knockdown of SLC2A1 was confirmed by RT-PCR. **(D, E)** CCK8 and clone formation assays were performed to assess cell viability and proliferation of A549 and H838 cells. **(F)** Transwell assay was performed to assess cell migration and invasion of A549 and H838 cells. **(G)** Wound healing effect of SLC2A1 in cell scratch assay: at 0 hour and 24 hours. **P < 0.01, ***P < 0.001.

To further elucidate the expression and functional implications of the riskscore, we initially conducted RT-qPCR analyses on nine genes in cell lines. In LUAD cells, the expression levels of SLC2A1, SFTPD, RRM2, CCNB1, CACNA2D2, SFTPB, DLGAP5, MYBL2, and HJURP were significantly higher compared to normal human lung cells, while PGC, CYP4B1, SFTPC, and CCNA2 showed marked decreases (**Figure 11**). Due to its pronounced importance and marked upregulation, SLC2A1 was selected for in-depth experimental validation. **Figures 12A, B** demonstrate that immunohistochemistry (IHC) revealed a marked overexpression of SLC2A1 in LUAD tissues relative to normal tissue. To explore SLC2A1’s specific role in LUAD, we engineered cell lines with stable SLC2A1 knockdown. Post-siRNA treatment targeting SLC2A1, RT-qPCR confirmed a significant reduction in its expression in these LUAD cells relative to controls (**Figure 12C**). Functional assays, including CCK8 and colony formation tests, demonstrated that SLC2A1 knockdown markedly inhibited LUAD cell proliferation (**Figures 12D, E**). Furthermore, Transwell assays showed significant reductions in cell migration and invasion following SLC2A1 suppression (**Figure 12F**), a finding that was supported by scratch wound healing assay (**Figure 12G**).

## Discussion

The incidence and mortality rates of LC are progressively increasing each year (41). Despite advancements in therapeutic drugs and treatment methods, managing LC recurrence or metastasis remains a significant challenge (42). Similar to other cancers, the variability in LUAD outcomes is primarily attributed to inherent molecular changes. The emergence of advanced sequencing technologies and bioinformatics has enabled a more comprehensive insight into the molecular changes in LUAD. Consequently, various novel risk stratification schemas have been developed, drawing on distinct altered molecules and forms. For example, Bhattacharjee and colleagues. classified LUAD into four subtypes by analyzing gene expression profiles, noting that these profiles could differentiate between primary and metastatic LUAD (43). In a similar study, Shibata and colleagues utilized genomic CNV data to classify LUAD into two subtypes via unsupervised clustering, noting that patients with EGFR mutations had reduced disease-free survival times (44). However, depending exclusively on one omics dataset offers a narrow perspective on the inherent molecular traits of tumors, and the insights gained from single-omics studies for tumor classification are somewhat constrained. Tumor heterogeneity is shaped by multiple omics layers, including the genome, epigenome, transcriptome, and proteome. Therefore, combining multi-omics data allows for the concurrent observation of tumor diversity across various dimensions and merges insights from several angles to more precisely determine tumor molecular classifications. This research employed ten clustering techniques to investigate the link between comprehensive data and OS results, identifying a cancer subtype that reflects the diversity of various omics in LUAD tissues, such as mRNA, lncRNA expression, CNVs, DNA methylation, and genetic mutations. The CS2 showed a more favorable outcome compared to CS1. Moreover, these two distinct CSs demonstrated significantly different molecular alteration landscapes and signaling pathway activations, resulting in varied immune statuses and biological behaviors.

Machine learning techniques are now recognized as effective tools for analyzing multi-omics data (45). To understand the molecular distinctions among different subtypes and enhance the clinical utility, we employed 10 widely-used machine learning algorithms to develop biomedical prognostic signatures using data from four multicenter LUAD cohorts. The efficacy of these signatures was evidenced by Kaplan–Meier, C-index, and ROC curve analyses, all of which demonstrated that the riskscore provided exceptional predictive performance across training, testing, and meta-cohorts. Additionally, when compared with clinical characteristics and 58 previously published LUAD signatures, the riskscore consistently showed superior accuracy in nearly all cohorts, underscoring its robustness.

In our research, we created a model consisting of 17 genes that accurately forecasts the outcome of LUAD. This model includes MYBL2, SLC2A1, CCNA2, HJURP, RRM2, CCNB1, TK1, and DLGAP5—eight genes whose elevated expression serves as hazard factors in LUAD. Predominantly, these genes contribute to the progression of LC. MYBL2, a key transcription factor within the Myb family, globally amplifies transcription, resulting in the significant dysregulation of target genes upon overexpression (46, 47). Xiong et al. reported that MYBL2 overexpression in LUAD correlates with advanced disease stages and reduced patient survival, facilitating LC cell proliferation and migration by upregulating NCAPH (48). CCNA2 is a cell cycle regulatory protein that controls the G1/S and G2/M transitions by binding to CDK1 and CDK2 (49, 50). It is markedly overexpressed in LUAD, correlating with poor prognosis (51, 52). Additionally, CCNA2 has been shown to foster abnormal tumor cell proliferation and epithelial-mesenchymal transition in NSCLC (53, 54). HJURP plays a critical role in DNA binding and phosphorylation, regulating chromosomal segregation and cell division (55). It is overexpressed in LC, enhancing NSCLC cell proliferation and metastasis through the inhibition of the Wnt/β-catenin pathway (56, 57). RRM2, encoding a subunit of ribonucleotide reductase, is essential for converting ribonucleotides to deoxyribonucleotides (58). Rahman et al. showed that RRM2 modification induces apoptosis by altering Bcl-2 expression in LC (59), and its low expression may predict the response to platinum-based chemotherapy in LC (60). Immunohistochemical analysis reveals that RRM2 is a strong prognostic marker in NSCLC (61). CCNB1 belongs to the cyclin family and plays a crucial role in the transitions between G2/M and metaphase/anaphase (62). MEOX1 inhibits LC cell progression by targeting the cell cycle checkpoint gene CCNB1 (63). Conversely, Bao et al. found that CCNB1 overexpression accelerates LC cell proliferation, migration, invasion, and cell cycle, whereas miR-139-5p can inhibit this effect (64). DLGAP5 is a microtubule-associated protein that supports the stable regulation of mitotic centromere fibers (65). Its overexpression correlates with poor outcomes in LUAD patients (66). Additionally, inhibiting DLGAP5 triggers cell cycle arrest and reduces the growth of NSCLC cells (67). In this study, SLC2A1 was selected for further functional validation due to its notably high differential expression among the evaluated genes in LUAD cell lines and its significant impact in our models. Our findings demonstrate that SLC2A1 enhances LUAD cell proliferation, migration, and invasion.

The interaction and co-evolution of the TIME and tumor cells are pivotal in driving tumor growth and progression, significantly influencing tumor sensitivity to treatments (68). Our research shows that in the high-risk group, many cancer-related pathways are significantly triggered, along with elevated TMB, TNB, and immune cell presence. Immunotherapy has dramatically altered the prognosis for patients with unresectable cancers (69). Although numerous drugs targeting immune checkpoints have received approval for cancer immunotherapy, including for LUAD, the lack of reliable biomarkers to predict treatment efficacy remains a challenge. Our analyses suggest that patients classified as high-risk may exhibit greater sensitivity to immunotherapy (70, 71). This prompted us to evaluate the utility of the riskscore in predicting patient responses to immunotherapy. Analysis using the TIDE and SubMap methods indicated that patients at high risk have a greater chance of benefiting from immunotherapy. Further analysis revealed that the riskscore was consistently increased in the response group compared to the non-response group within immunotherapy cohorts, supporting our initial findings. Therefore, the riskscore could serve as a predictive marker for immunotherapy efficacy, with high-scoring LUAD patients potentially achieving greater benefits from such treatments.

The concurrent use of chemotherapy and immunotherapy is a focal point of current cancer research, as it leverages the immune-modulating effects of immunotherapy to mitigate the immune damage from chemotherapy, resulting in a synergistic antitumor response (72, 73). For the high-risk group, particular chemotherapy drugs were pinpointed to enhance treatment plans for LUAD. Paclitaxel, cabazitaxel, and epothilone, commonly used for treating advanced non-small cell lung cancer, work by stabilizing microtubules, thereby preventing cell division and inducing apoptosis in cancer cells (74–76). Furthermore, SB743921, a highly effective next-generation KSP inhibitor, has shown tumor-fighting capabilities in multiple types of cancer (77, 78). A phase I clinical trial indicated that a cholangiocarcinoma patient showed a partial response to SB743921 treatment after 7 months, which lasted until the disease advanced approximately 12 months later (79). Daunorubicin is well recognized for its ability to intercalate into DNA and disrupt the DNA replication process, which constitutes its primary mechanism for exerting anticancer effects. As a first-line treatment for leukemia, daunorubicin is commonly administered in combination with other chemotherapeutic agents, such as cytarabine (80). In the case of LUAD, the combination of dendrosomal curcumin and daunorubicin notably diminished tumor progression, triggered cell death, and lowered cell movement in A549 cells, with effects varying according to dosage and duration (81). Our analysis of drug sensitivity revealed that individuals in the high-risk group showed greater responsiveness to the specified chemotherapy drugs, implying that patients with elevated riskscores could gain significant benefits from these therapies.

However, several limitations of the current study should be noted when interpreting our findings. Firstly, the biomarkers in this study were mainly derived from patient classification based on multi-omics data, which can reduce the impact of tumor heterogeneity on tumor classification. More comprehensive multi-omics data could potentially classify patients more accurately and lead to better biomarkers. As the multi-omics data available in this study is not entirely comprehensive, the classification results obtained may not be optimal, with potential to include more omics data (for example, lacking proteomics and metabolomics data) to achieve greater accuracy in analysis. Secondly, the molecular subtypes and model construction for LUAD in this study were based on a retrospective cohort. Retrospective studies are typically based on historical records, and the data often come with limitations such as information bias, limited representativeness, and the inability to directly predict future trends. In comparison, prospective studies can mitigate bias by setting standards, collecting a wider range of samples and variables, and gathering dynamic data after testing, thereby improving the generalizability, accuracy, and applicability of the results. Therefore, confirmation through prospective studies is necessary. Besides, although this research provided initial validation of SLC2A1’s role in LUAD, genes express their effects through multiple mechanisms, including transcriptional regulation, epigenetics, tumor microenvironment, and mutation patterns. Therefore, we have not yet clarified how SLC2A1 exerts its oncogenic effects in LUAD, and further experiments are needed to verify the potential mechanisms. Therefore, we plan to gather a sufficient number of LUAD patient samples to reconstruct the same model as in this study and follow up to assess the model’s applicability and reliability in the future. Meanwhile, we will perform extensive multi-omics testing and analysis (encompassing proteomics and metabolomics) on these collected patient samples to discover more effective new biomarkers. Furthermore, we will also conduct further research on the transcriptional and pathway molecular mechanisms of SLC2A1 in LUAD to clarify the details of SLC2A1’s oncogenic role in this disease.

## Conclusion

In conclusion, we have successfully classified LUAD into two distinct subtypes by integrating various omics data. These variants are strongly associated with variations in patient outcomes, features of the tumor microenvironment, and molecular signatures. Our discoveries improve the comprehension of LUAD’s diversity and its fundamental pathological processes. This novel categorization method has the potential to greatly enhance precision medicine by guiding the creation of specialized clinical tactics for radiotherapy and immunotherapy in patients with LUAD.

## Data Availability

The datasets presented in this study can be found in online repositories. The names of the repository/repositories and accession number(s) can be found in the article/**Supplementary Material**.
