# Supplementary material for: Multi-omics characterization and machine learning of lung adenocarcinoma molecular subtypes to guide precise chemotherapy and immunotherapy
Source: Front Immunol. 2024 Nov 28;15:1497300. doi: 10.3389/fimmu.2024.1497300 (PMC11634853; doi:10.3389/fimmu.2024.1497300)
Supplement: Supplementary file 1 [file DataSheet1.pdf]

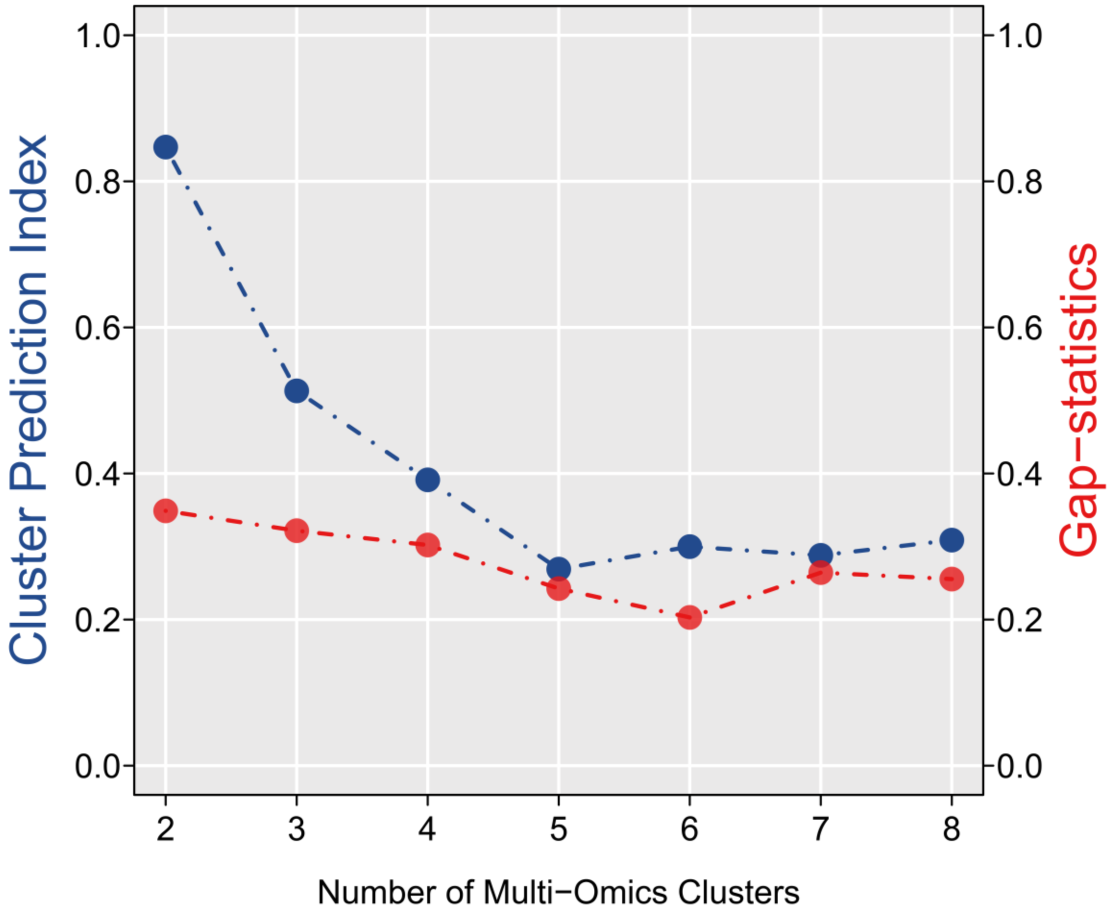

Supplementary Figure 1 The cluster prediction index and gap statistical analysis for the evaluation of multiomics clusters.

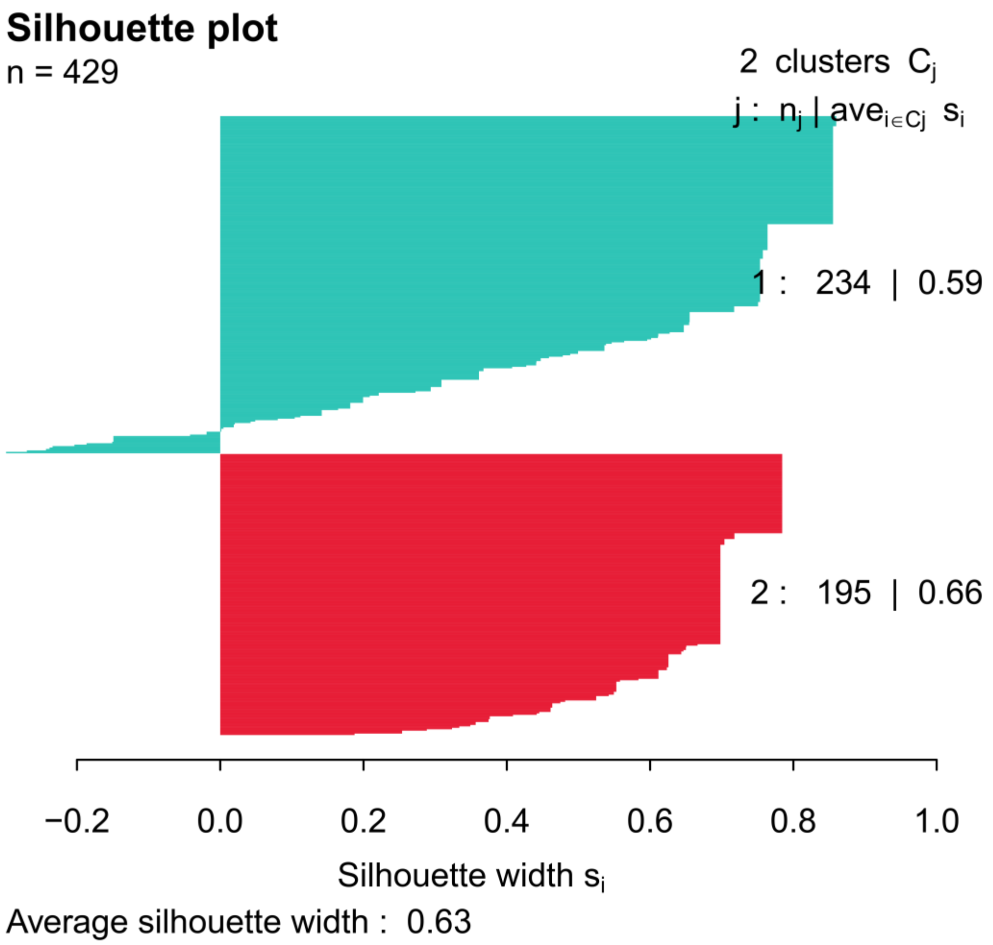

Supplementary Figure 2 The subgroup sample similarity was evaluated through the computation of silhouette scores for each subtype.

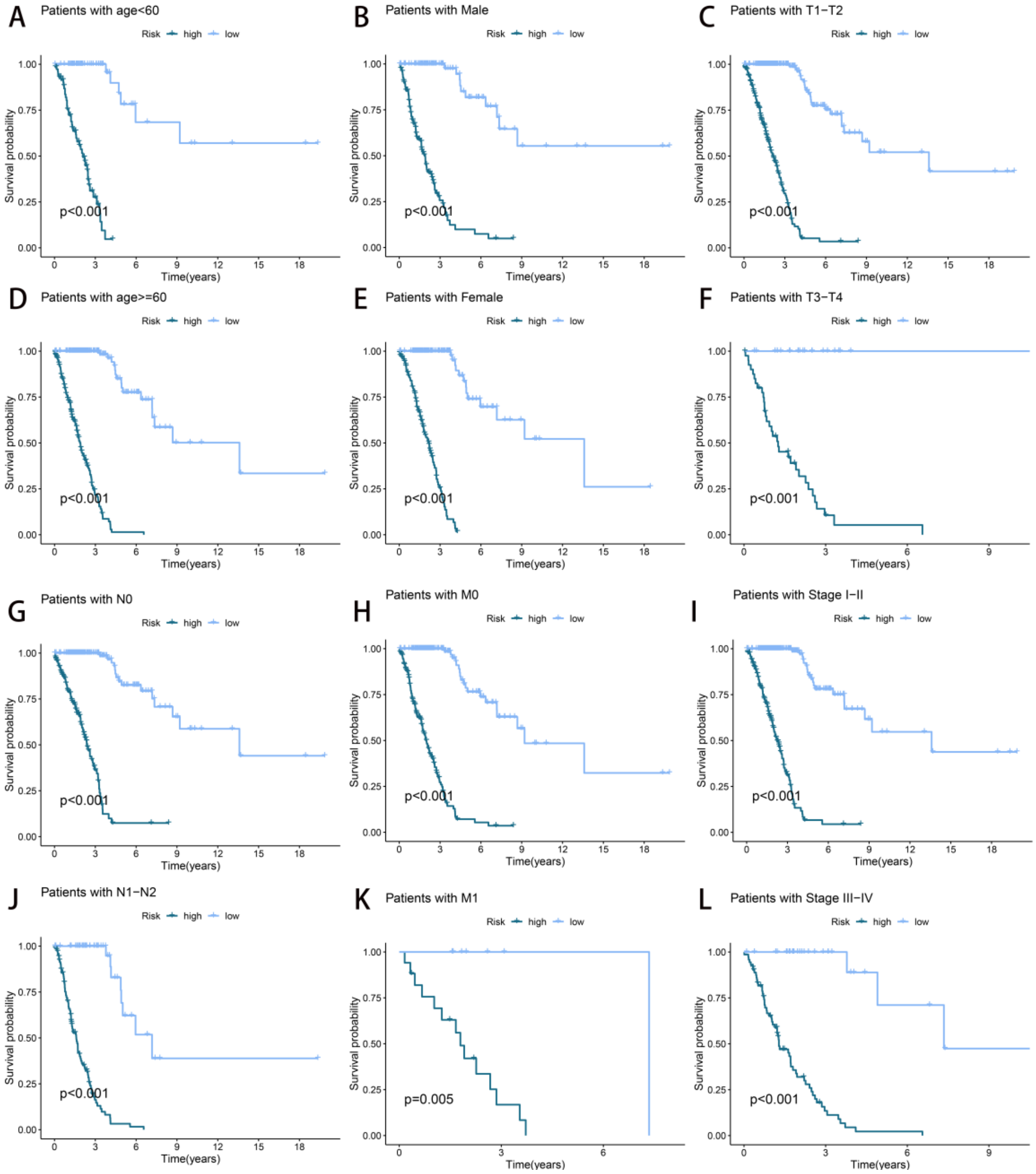

Supplementary Figure 3 The survival curves of the Cov-2S stratified by age, gender, T, N, M and stage. **(A)** ≥60 years, **(B)** male, **(C)** T1-2, **(D)** <60 years, **(E)** female, **(F)** T3-4, **(G)** N0, **(H)** M0, **(I)** stage1-2, **(J)** N1-2, **(K)** M1, **(L)** stage3-4.

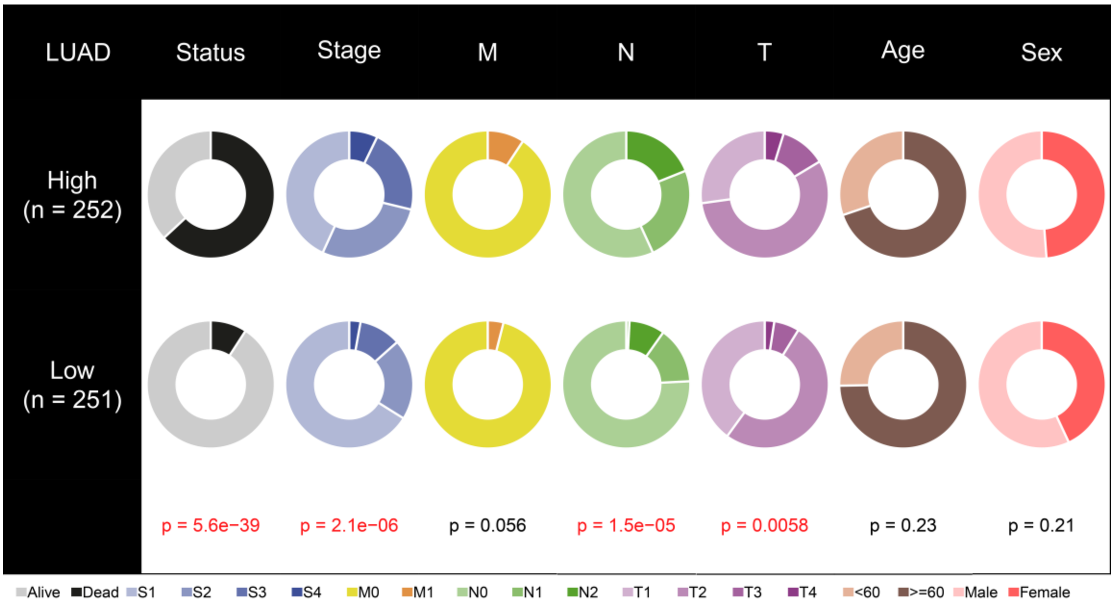

Supplementary Figure 4 The circular pie chart for the proportion difference of clinical indices.

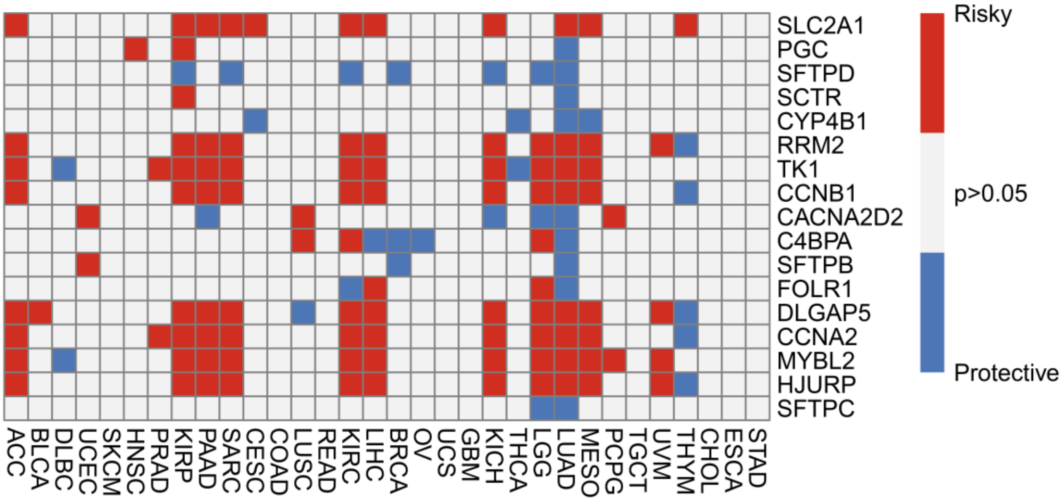

Supplementary Figure 5 The heatmap illustrates the prognostic capability of riskscore genes across pancancer.

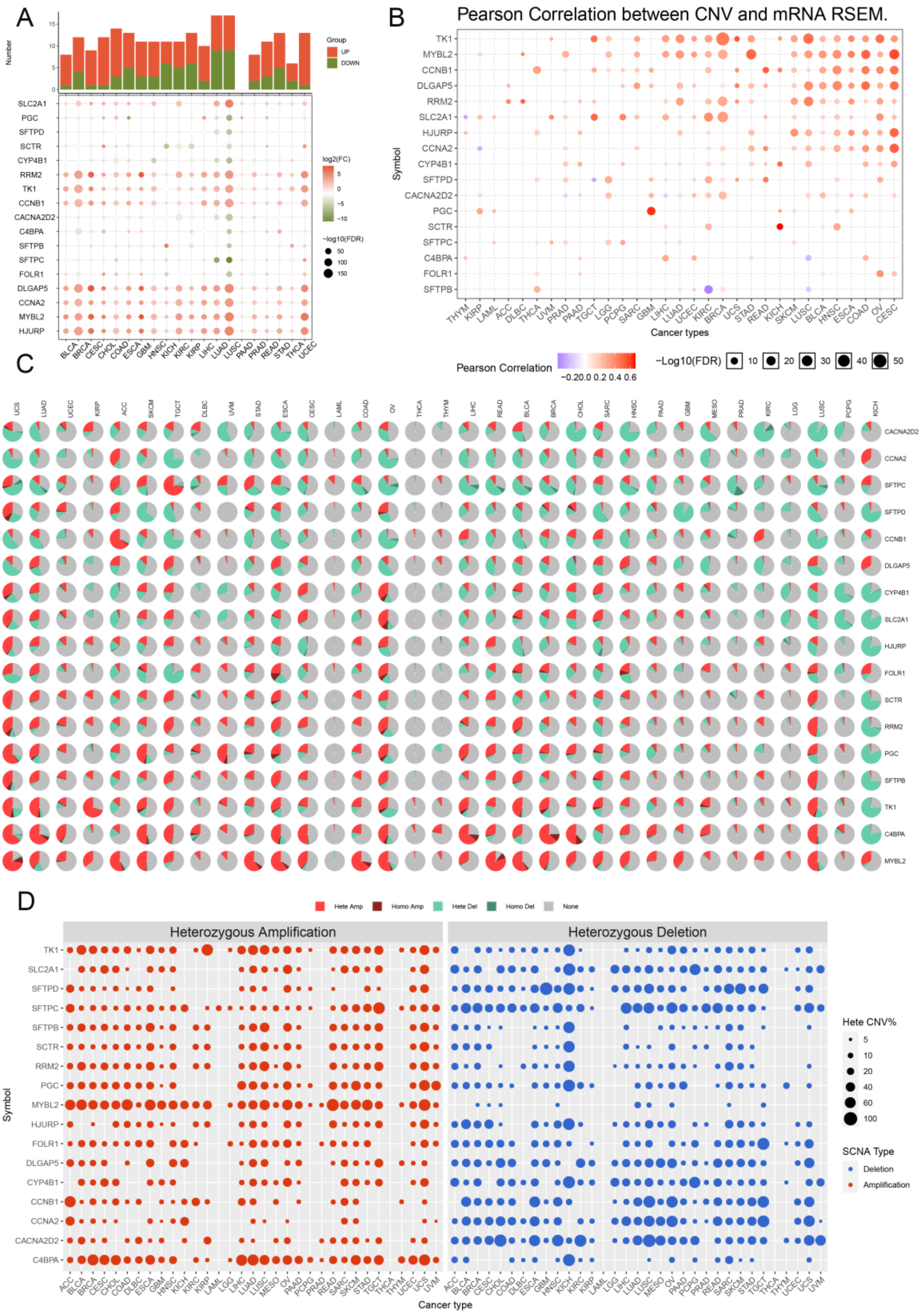

Supplementary Figure 6 Differential expression analysis and CNV-related analysis of riskscore genes. (A) The heatmap depicts the expression variance of riskscore genes between normal and tumor tissues across pancancer. **(B)** Correlation analysis of CNV with mRNA expression levels of riskscore genes. **(C)** The pie chart illustrates Heterozygous/Homozygous CNV distribution for individual riskscore genes across pancancer. **(D)** The distribution of heterozygous and homozygous CNV among hub

genes within the riskscore across pancancer.

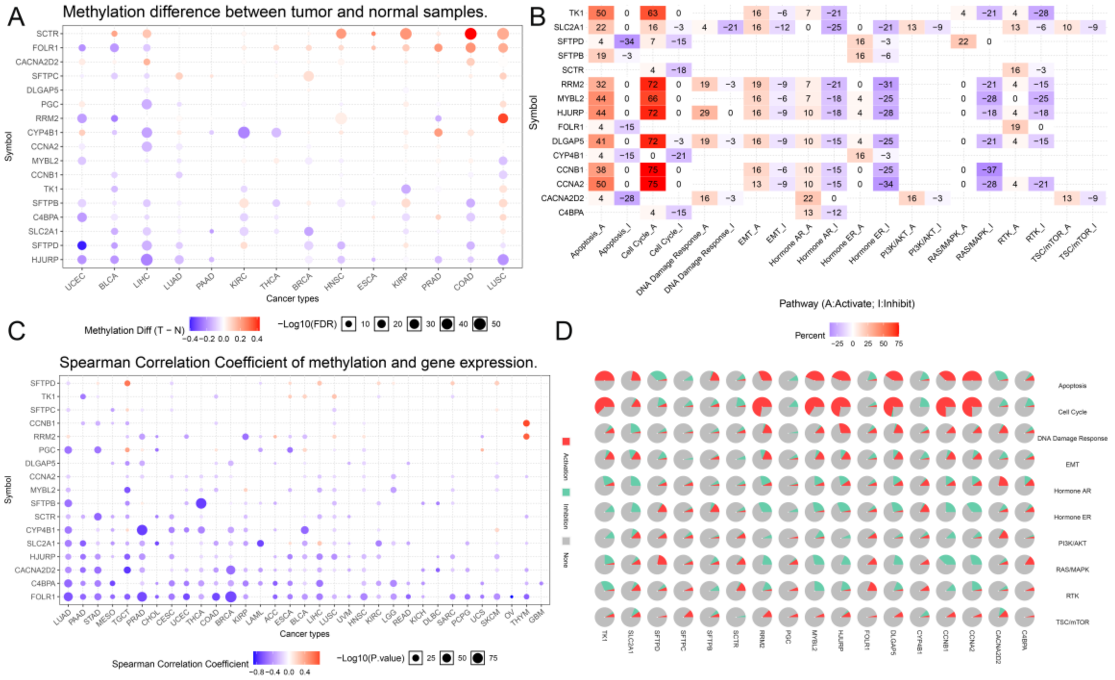

Supplementary Figure 7 Methylation-related analysis and pathway analysis of riskscore genes. (A) Differential methylation expression analysis of riskscore genes. **(B)** Correlation analysis of methylation with mRNA expression levels for the riskscore genes. **(C-D)** The heatmap and pie chart depict the activation and repression status of riskscore genes on associated pathways.

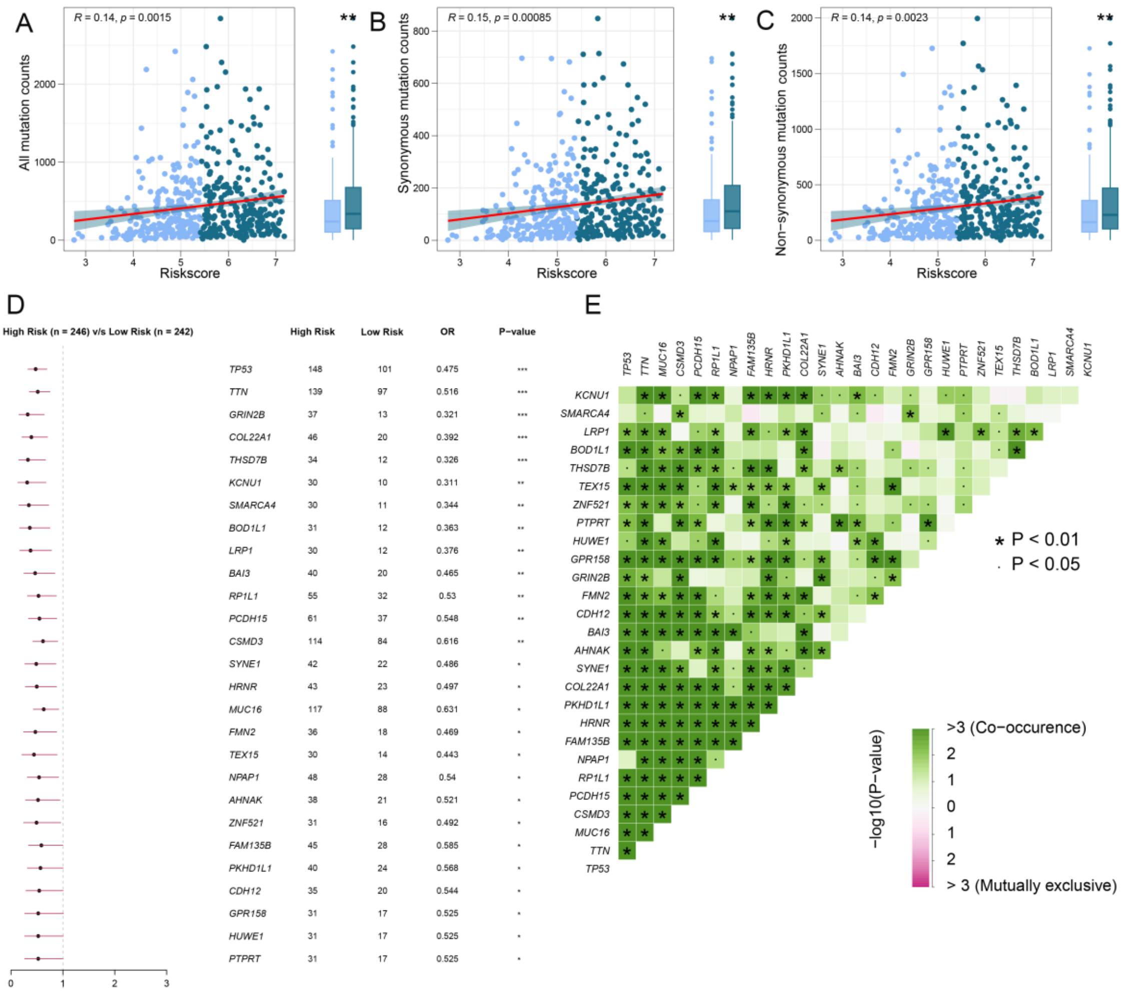

Supplementary Figure 8 Correlation analysis between mutations and riskscore. (A-C) Association between all mutation counts, synonymous mutation counts, nonsynonymous mutation counts, and riskscore and their distribution in the riskscore groups. **(D)** Differentially mutated genes between high and low risk groups are displayed as a forest plot. **(E)** Interaction effect of genes mutating differentially in patients in the risk groups.

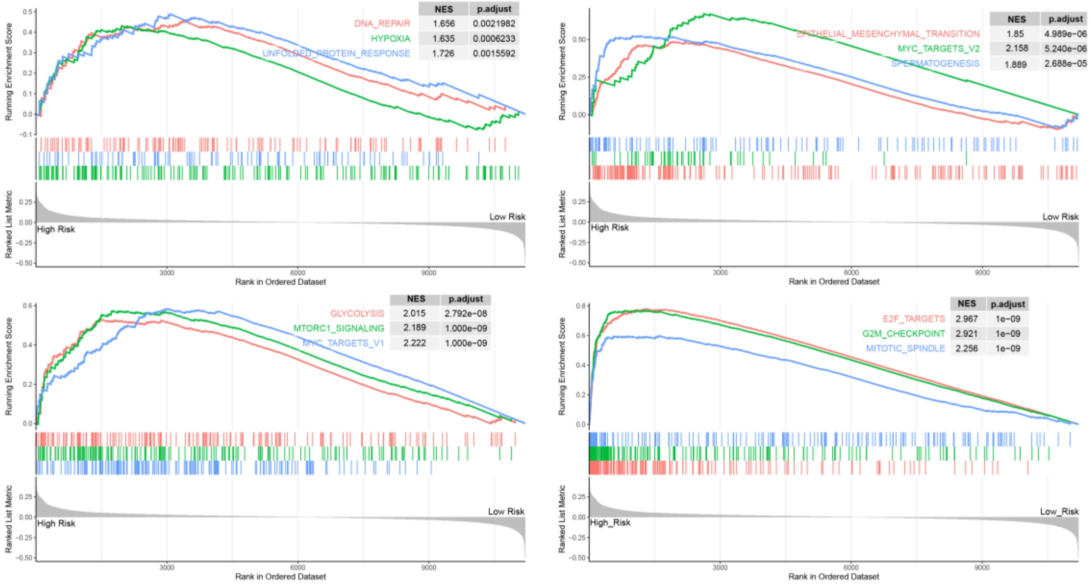

Supplementary Figure 9 The GSEA results for the 12 overlapping upregulated hallmark pathways in terms of the high risk group.
