# Supplementary material for: Multi-omics characterization and machine learning of lung adenocarcinoma molecular subtypes to guide precise chemotherapy and immunotherapy
Source: Front Immunol. 2024 Nov 28;15:1497300. doi: 10.3389/fimmu.2024.1497300 (PMC11634853; doi:10.3389/fimmu.2024.1497300)
Supplement: Supplementary file 2 [file DataSheet2.pdf]

Supplementary Methods

Generation of Riskscore

To construct a robust and precise consensus model (RiskScore), we utilized an ensemble approach involving ten machine learning algorithms: random survival forest (RSF), elastic network (Enet), Lasso, Ridge, stepwise Cox, CoxBoost, partial least squares regression for Cox (plsRcox), supervised principal components (SuperPC), generalized boosted regression modeling (GBM), and survival support vector machine (survival-SVM). Key algorithms, including Lasso, stepwise Cox, CoxBoost, and RSF, were effective in feature selection. These models were combined to create a unified predictive framework using 101 unique algorithm combinations, each developed within a leave-one-out cross-validation (LOOCV) scheme. The initial signature was discovered using TCGA-LUAD data. For RSF, we employed the "randomForestSRC" package, optimizing its ntree and mtry parameters via a grid search in the LOOCV setting, selecting the parameter pair with the highest C-index. Enet, Lasso, and Ridge were implemented with the "glmnet" package, where the regularization parameter λ was determined through LOOCV, and the L1-L2 trade-off parameter α was varied systematically from 0 to 1 in intervals of 0.1. The stepwise Cox model, realized through the "survival" package, utilized the Akaike information criterion (AIC) and explored “both,” “backward,” and “forward” search directions. CoxBoost was implemented using the CoxBoost package, with the optimal penalty set via LOOCV's optimCoxBoostPenalty function, while the number of boosting steps was fine-tuned with cv.CoxBoost, and the model’s dimension was established through the main CoxBoost routine. For plsRcox, we used the "plsRcox" package, determining the necessary number of components through cv.plsRcox to fit a partial least squares regression generalized linear model. SuperPC, sourced from the "superpc" package, was applied to extract dominant directions of variation. GBM modeling was executed with LOOCV to minimize cross-validation error through the cv.gbm function, followed by configuration with gbm. Lastly, the survival-SVM model, established with the "survivalsvm" package, incorporated censoring through a regression approach, which imposed inequality constraints to address the challenges specific to censored data.
